# The interaction between luminance polarity grouping and symmetry axes on the ERP responses to symmetry

**DOI:** 10.1017/S0952523824000075

**Published:** 2024-12-16

**Authors:** Benjamin Dering, Damien Wright, Elena Gheorghiu

**Affiliations:** 1 Department of Psychology, University of Stirling, Stirling, FK9 4LA, Scotland, United Kingdom; 2 Patrick Wild Centre, Division of Psychiatry, Royal Edinburgh Hospital, Edinburgh, EH8 9XD Scotland, United Kingdom

**Keywords:** symmetry, luminance polarity, grouping, sustained posterior negativity, event-related potentials

## Abstract

Symmetry is a salient visual feature in the natural world, yet the perception of symmetry may be influenced by how natural lighting conditions (e.g., shading) fall on the object relative to its symmetry axis. Here, we investigate how symmetry detection may interact with luminance polarity grouping, and whether this modulates neural responses to symmetry, as evidenced by the Sustained Posterior Negativity (SPN) component of Event-Related Potentials (ERPs). Stimuli were dot patterns arranged either symmetrically (reflection, rotation, translation) or quasi-randomly, and by luminance polarity about a grouping axis (i.e., black dots on one side and white dots on the other). We varied the relative angular separation between the symmetry and polarity-grouping axes: 0, 30, 60, 90 deg. Participants performed a two interval-forced-choice (2IFC) task indicating which interval contained the symmetrical pattern. We found that accuracy for the 0 deg polarity-grouped condition was higher compared to the single-polarity condition for rotation and translation (but not reflection symmetry), and higher than all other angular difference (30, 60, 90) conditions for all symmetry types. The SPN was found to be separated topographically into an early and late component, with the early SPN being sensitive to luminance polarity grouping at parietal-occipital electrodes, and the late SPN sensitive to symmetry over central electrodes. The increase in relative angular differences between luminance polarity and symmetry axes highlighted changes between cardinal (0, 90 deg) and other (30, 60 deg) angles. Critically, we found a polarity-grouping effect in the SPN time window for noise only patterns, which was related to symmetry type, suggesting a task/ symmetry pattern influence on SPN processes. We conclude that luminance polarity grouping can facilitate symmetry perception when symmetry is not readily salient, as evidenced by polarity sensitivity of early SPN, yet it can also inhibit neural and behavioral responses when luminance polarity and symmetry axes are not aligned.

## Introduction

**Figure 1.**
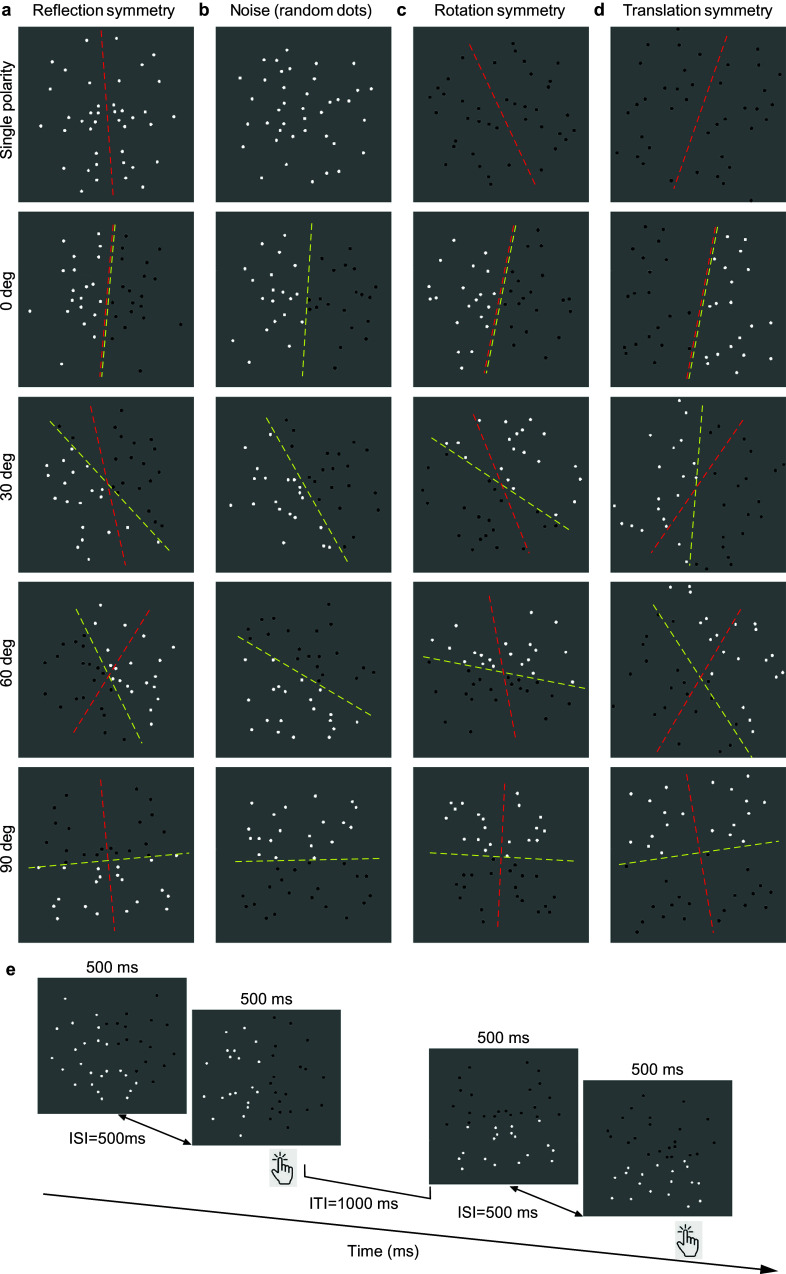
Example (a) reflection-symmetric, (b) random (or noise), (c) rotation-symmetric and (d) translation-symmetric patterns made of single-polarity (either white or black; see top panels) and polarity-grouped elements. The angle between the symmetry (red) and polarity-grouping (yellow) axes was either 0, 30, 60, and 90 deg (top to bottom panels). The red and yellow lines are for illustration purpose only. (e) Example sequence of trials for the two interval-forced-choice procedure used to measure reflection-symmetry detection. On each trial, a reflection-symmetric stimulus was randomly presented for 500 ms in one of the two intervals while the other interval contained a noise pattern made of quasi-random position dots presented for 500 ms. The inter-stimulus interval (ISI) was 500 ms and the inter-trial interval (ITI) was 1000 ms (see text for details).

Every image of the natural world that contains a pattern must also have some degree of symmetry. In nature, there are different forms of symmetry observable within a single object. For example, many flowers like daisies can contain both whole flower symmetry (e.g., rotation or reflection) and local symmetries (e.g., reflection symmetry of petals). When we view these objects in natural lighting conditions, shadows (i.e., luminance-defined features) can fall on them at different angles relative to their axes of symmetry. How do position symmetry and luminance polarity grouping interact in the perception of object symmetry? In this communication, we answer the above question by exploring the interaction between different symmetry types (reflection, rotation, translation – see Figure1a,c,d) and luminance polarity axes, using psychophysical methods and a reliable marker of symmetry detection in the brain, the Sustained Posterior Negativity (SPN) difference wave of Event-Related Potentials (ERPs).

Several studies of symmetry perception have compared sensitivity between reflection, rotation, and translation symmetry using a variety of measures (detection and discrimination thresholds, reaction times, electroencephalography (EEG), functional magnetic resonance imaging (fMRI), type of stimuli (dot and line patterns, closed shapes), and tasks (detection, discrimination, type of symmetry identification) – for reviews see: Wagemans (1995, 1997), Treder (2010), and Bertamini et al. (2018). Converging evidence from these diverse approaches suggests greater sensitivity to reflection compared to other forms of symmetry (Wagemans, 1995), although rotation symmetry is frequently occurring in nature. More often reflection symmetry when encountered in nature is defined along one axis (often oriented vertically), while rotational-symmetric patterns have multiple axes, and thus remain invariant to rotation (Palmer and Hemenway, 1978; Kahn and Foster, 1981; Royer, 1981; Zimmer, 1984; Kahn and Foster, 1986; Jennings and Kingdom, 2017), for example, a spinning pinwheel. Further, less studied but equally relevant is translation symmetry which occurs in the context of repeating/regular patterns (Corballis and Roldan, 1974; Bruce and Morgan, 1975; Tyler and Chang, 1977; Kahn and Foster, 1986; Baylis and Driver, 1994; van der Helm and Treder, 2009; Bertamini, 2010; Makin et al., 2014), such as wallpaper patterns that often contain other forms of symmetry such as reflection and rotation (Kohler et al., 2016; Alp et al., 2018; Kohler and Clarke, 2021). This may explain the apparent ease of reflection symmetry detection in contrast to rotation and translation symmetry. However, the importance of visual attributes such as *luminance polarity* in the analysis of different types of symmetry remains controversial.

A number of studies have highlighted the disrupting effect of luminance polarity changes on symmetry detection using positionally symmetric elements mismatched in luminance polarity across the symmetry axis, demonstrating that symmetry mechanisms are sensitive to luminance polarity (Zhang and Gerbino, 1992; Wenderoth, 1996; Brooks and van der Zwan, 2002). On the other hand, other studies suggest that luminance polarity does not affect reflection symmetry detection (Rainville and Kingdom, 1999; Saarinen and Levi, 2000; Rainville and Kingdom, 2002; Mancini et al., 2005; Gheorghiu et al., 2016). Yet, this type of polarity-mismatch arrangement could be argued to be less ecologically applicable since natural shading predominantly occurs over a larger area, rather than patch-wise. When grouping by luminance polarity is between two symmetric halves, e.g., one half black, one half white, reflection symmetry detection has also been shown to be disrupted (Zhang and Gerbino, 1992; Wenderoth, 1996), suggesting that grouping by polarity does not improve symmetry detection. In contrast, single-polarity symmetric patterns compared to antisymmetric patterns (black–white symmetric pairs not polarity-grouped across symmetry axis) have been found to elicit similar levels of performance (Wenderoth, 1996) when the patterns are of low dot densities. This has led to the suggestion that there is no relationship between symmetry detection and the degree of luminance polarity correlation across the symmetry axis. To the best of our knowledge, previous studies have only examined the situation in which polarity-grouping and symmetry axes coincide (Zhang and Gerbino, 1992; Wenderoth, 1996; Wright et al., 2018).

There are several explanations for why performance is the same in single-polarity and antisymmetric patterns in low dot-density displays. One comes from computational models of reflection symmetry, where two general approaches have been implemented. A top-down approach relying upon complex grouping rules from which reflection symmetry is subsequently extracted (Wagemans et al., 1993; Labonte et al., 1995; Osorio, 1996) or a bottom-up approach that uses early spatial mechanisms such as oriented filters to compute reflection symmetry (Dakin and Watt, 1994; Osorio, 1996; Rainville and Kingdom, 2000). Critically, models of symmetry based on early spatial mechanisms such as oriented filters, e.g., filter-rectify-filter models (Wagemans et al., 1993; Dakin and Watt, 1994; Labonte et al., 1995; Tyler and Hardage, 1995; Rainville and Kingdom, 2000), suggest that second-order channels, which integrate first-order inputs, are insensitive to luminance polarity, and therefore are *position-symmetry sensitive only.* A second explanation for detecting symmetry in antisymmetric and single-polarity patterns at the same performance level in low-density displays relies on the involvement of selective attentional mechanisms (registering the positional symmetry of individual dots differing in luminance polarity) which cannot operate in higher-density displays (Mancini et al., 2005). However, no model has so far taken into account how changes in luminance polarity grouping affect detection. Further, these models have been separately developed for each type of symmetry with more focus being placed upon reflection symmetry detection.

With regard to neuroimaging studies of symmetry detection, these highlight an ERP difference wave, termed the SPN, which is responsive to the presence of symmetry, and onsets from 250 ms onwards (for a review see: Bertamini et al. (2018), Bertamini and Makin (2014)). The SPN response is thought to be automatic and task-independent, since it can be elicited also from orthogonal tasks which do not rely upon direct symmetry detection (Jacobsen and Hofel, 2003; Hofel and Jacobsen, 2007; Wright et al., 2017), hence the SPN is thought to reflect the salience of symmetry in a stimulus. The SPN has also been shown to scale with the amount of symmetry in the stimulus, such as 100% versus 50% symmetry, which reflects the ease of symmetry perception in the stimulus (Makin et al., 2013; Wright et al., 2018; Makin et al., 2020). It is important to note that the link between stimulus duration and SPN duration is unknown, although some evidence suggests that SPN can persist beyond stimulus offset (Bertamini et al., 2019). Furthermore, the SPN has often been linked to extrastriate cortex activity (Norcia et al., 2002), as evidenced from source localization (Makin et al., 2012), yet the majority of EEG studies focus the analysis on two electrodes of interest PO7 and PO8 (for a review see: Makin et al. (2022)). The SPN has also been shown to be the largest in amplitude for reflection symmetry, while rotation and translation symmetry show a reduced SPN response (Makin et al., 2013). It is worth noting that in some studies examining SPN, symmetry sensitivity has also been found to occur earlier than SPN, for example, in the P1 for rotational symmetry (Makin et al., 2012) or the later N1 (Makin et al., 2022), suggesting that the neural responses to symmetry might develop earlier than the onset of SPN differences.

The effects of luminance polarity on SPN responses have been studied under two approaches: one in which polarity changes across stimuli (and time), and second when the polarity changes within the stimuli either across the symmetry axis or within the pattern. Firstly, a single study employing a priming paradigm using a temporal succession of symmetric patterns, which were either the same polarity (all white or all black) or alternating from white to black or vice-versa, showed comparable SPN responses to the same and alternating polarity sequences (Makin et al., 2021), suggesting that luminance increments/decrements of patterns do not affect the strength of the symmetry response. However, when using antisymmetric patterns in which elements are mismatched in polarity across the symmetry axis, the SPN response was found to be reduced in comparison to single-polarity and/or polarity-grouped symmetric patterns (Makin et al., 2016; Wright et al., 2018). In addition, when comparing antisymmetric with polarity-grouped antisymmetric patterns, Wright et al. (2018) found no differences in SPN response between the two types of antisymmetric patterns.

Our own evidence suggests that the SPN can be divided topographically into two separate components, an early component involved in the detection of a stimulus gestalt/ pattern perception (e.g., polarity-grouping) and a late component responsive to the presence of symmetry (Wright et al., 2018). We suggested that the late component of the SPN response may explain the prolonged nature of this difference wave. In Wright et al. (2018), we examined polarity-grouping effects using fully antisymmetric patterns in which either the halves were black/white grouped or made of mixed black/white pairs, yet critically, any polarity-grouping axis was always aligned with the symmetry axis. Hence, we further explore here how the angular difference between symmetry and luminance polarity grouping axes affects symmetry detection, utilizing the SPN as a reliable index of symmetry processing. Furthermore, we aim to examine the relationship between the luminance polarity grouping and symmetry axes by varying the relative angle between the two, for three types of symmetry (reflection, rotation, and translation).

In the current study, we aim to examine how relative changes between symmetry and luminance polarity axes affect symmetry detection and neural responses to symmetry for three commonly observed types of symmetry – vertical reflection, rotation, and translation symmetry. We used both single and polarity-grouped patterns, and for polarity-grouped patterns only, varied the relative axes between vertical symmetry and luminance polarity grouping (0, 30, 60, 90 deg). Note that in order to avoid always using an absolute vertical axis as a reference point (i.e., participants would focus on the vertical dimension only) we used a random amount of positional jitter of the symmetry axis within a +/−30 deg range; however, the relative angle was always in relation to the symmetry axis. For all three symmetry types, we predict that luminance polarity grouping, when aligned with the vertical axis of symmetry (0 deg), will produce similar performance and comparable SPN responses to the single-polarity condition, within each type of symmetry. Further, we predict reduced performance and decreased SPN amplitude for 30 and 60 deg compared to 0 and 90 deg angular differences. This is because the 90 deg condition is fully symmetric in terms of both position and polarity matching (but upper/ lower halves have different polarity), and the 0 deg condition is fully antisymmetric (i.e., individual symmetric pairs are all mismatched in polarity, but stimulus halves are grouped by polarity; see Figure 1). Based upon our previous findings on SPN polarity-grouping effects in symmetric patterns (Wright et al., 2018), we predict that the processes occurring during the early SPN time window might reflect polarity-grouping effects even in noise only patterns (i.e., single-polarity versus polarity-grouped noise). In addition, since Wright et al. (2018) separated the SPN into topographically distinct components, we will further examine the topographical time course of the SPN using ERPs and microstates (see Supplementary Materials – Appendix A), to gain insight into the time windows related to polarity- grouping phenomena and symmetry.

## Methods

### Participants

Twenty-six participants, who were all naive with regard to the experimental aims, took part in this study. All participants had normal or corrected-to-normal vision. Two participants were removed from all analyses due to lack of discernible ERP signal (see section “Procedure – EEG recording and analysis”), leaving a total of 24 participants. All participants gave their written informed consent prior to participating in the study and were treated in accordance with the Declaration of Helsinki (2008, version 6). All research procedures were approved by the Research Ethics Committee, University of Stirling, UK.

### Stimuli – generation and display

Stimuli were dot patterns presented on a calibrated, gamma-corrected 20” ViewSonic Graphics Series G225f cathode ray tube (CRT) monitor (ViewSonic, Brea, CA, USA), running at 60 Hz frame rate and with spatial resolution of 1024 × 768 pixels. All stimuli were presented in the center of the monitor on a mid-grey background with mean luminance of 65.5 cd/m^2^. The experimental setup was placed in a dark, sound-attenuated room. Viewing distance was 60 cm.

The stimuli had a diameter of 12 deg and were made of 40 achromatic Gaussian blobs with a standard deviation of 0.08 deg and a Gaussian size standard deviation factor of 5. The dot patterns were arranged either symmetrically (100% position symmetry) or quasi-randomly. We used three types of one-fold symmetry: reflection, rotation, and translation symmetry (Figure 1a,c,d). The location of the symmetry axis was randomly jittered from trial to trial within +/−30 deg jitter range. For each type of symmetry, there were two luminance polarity conditions: (1) *single-polarity symmetric patterns* in which all position-symmetric elements had the same luminance polarity, either black or white (Figure 1, top panels); (2) *polarity-grouped* in which position-symmetric dots were of opposite luminance polarity across the symmetry axis, but with one luminance polarity on one side of the symmetry axis (i.e., either white or black dots) and opposite luminance polarity on the other (i.e., either black or white). We varied the relative angular separation between the symmetry axis and polarity-grouping axis: 0, 30, 60, 90 deg (Figure 1). As with the symmetric patterns, we used two types of quasi-random dot (or noise) patterns (a) single-polarity noise in which all dots were either white or black, and (b) polarity-grouped noise in which half of the random pattern was of one luminance polarity (either white or black) and the other was of opposite polarity (either black or white) – see Figure 1b. Note that the polarity-grouping axis for the noise was not always vertical but the noise had an absolute orientation angle of 0, 30, 60, 90 deg relative to the vertical. This was done in order to avoid participants making a decision based solely upon a single orientation (0 deg) of the polarity-grouping axis in the two interval-forced-choice (2IFC) task.

### Procedure – 2IFC

A 2IFC procedure was used to measure symmetry detection. On each trial, a stimulus corresponding to one of the symmetric conditions was randomly presented in one of the two intervals while the other interval contained a noise pattern made of quasi-random position dots (i.e., the null interval) – see Figure1e. For the single-polarity conditions, we used a single-polarity noise pattern, i.e., all black or all white dots, while for the polarity-grouped conditions, we used noise patterns that were half-white and half-black. However, for the purpose of analysis, we use the terms reflection-noise, rotation-noise, and translation-noise to identify the specific noise stimuli associated with that symmetry type. From trial to trial, the location of the symmetry axis was randomly jittered within a 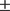 30 deg range. In both intervals, the orientation of the polarity-grouping axis for the symmetric and noise patterns was identical. Each pattern was presented for 500 ms with an inter-stimulus interval of 500 ms. The task of the participants was to indicate which interval contained the symmetric pattern by responding via a key press. The inter-trial interval (ITI) was 1000 ms. Each relative angular separation between symmetry and polarity- grouping axes condition was presented 100 times with stimulus conditions presented in random order. The experiment was blocked by the type of symmetry, i.e., reflection, rotation, and translation, in order to reduce uncertainty. Given the five stimulus conditions (i.e., single-polarity, 0, 30, 60, and 90 deg relative angular separation), this resulted in a total of 500 trials for each type of symmetry. The experiments were further divided into blocks of 125 trials to allow the participant regular breaks and for the electrodes to be checked.

### Procedure – EEG recording and analysis

Electroencephalograms were recorded via a SynAmps 2 amplifier and Scan 4.5 software (Neuroscan Inc., El Paso TX, USA). Four external channels recorded bipolar and horizontal and vertical electrooculograph (EOG) signals. Raw EEG signals were recorded from the scalp using a sampling rate of 1 kHz from 64 Ag/AgCl electrodes positioned according to the extended 10–20 system, with an online reference of CZ electrode. The electrodes recording impedances were kept below 5KΩ. The EEG was filtered offline with a band-pass filter between 0.1 and 30 Hz (12dB/octave and 96 dB/octave slope, respectively). Blink artifacts were corrected using a model blink artifact computed for each individual based upon the method of Gratton et al. (1983)). Trials exceeding ±125 μV in any epoch were discarded. All averages were baseline-corrected using a pre-stimulus baseline of 100 ms. Grand averages were calculated after re-referencing individual participant ERPs to the common average reference. Participants whose data showed irretrievable noise contamination or a significant loss of channels, leading to no discernible ERP signal, were removed from the analysis, resulting in the removal of two participants from the EEG analysis. This left a total of 24 participants in the analysis.

*Analysis* was performed on the grand averages of electrodes PO7 and PO8. These electrodes were chosen as they were consistent with electrode selections used in previous symmetry research (Bertamini and Makin, 2014; Makin et al., 2014; Wright et al., 2017; Wright et al., 2018). P1 and N1 peak amplitude were determined for each condition individually before calculation of mean amplitude in a 40 ms time window around each peak (P1 mean amplitude time windows: 65–115 ms; N1: 141–194 ms). This was done to be able to analyze P1 and N1 amplitude differences separately from latency. The SPN was calculated as the ERP difference wave from 250 ms to 600 ms after stimulus onset between symmetric and quasi-random patterns with corresponding orientation of the polarity-grouping axes – for example, symmetry pattern of 60 deg relative angular difference between polarity and symmetry axes minus noise pattern of 60 deg polarity axis orientation. We chose to analyze the SPN up to 600 ms after stimulus onset, even though the SPN could extend beyond this time-point, in order to avoid any contamination from changes due to offset potentials. In addition, we analyzed ERP changes produced by noise patterns only over the SPN time window. Because noise stimuli varied with the change in polarity-grouping angle (0, 30, 60, 90 deg; see Figure1b) and therefore were not equivalent baselines, we also analyzed the SPN for each symmetry condition relative to a single noise baseline per symmetry type (e.g., reflection-symmetry pattern of 60 deg relative angular differences with a 0 deg reflection-noise polarity axis).

Given our previous research (Wright et al., 2018) suggesting that the SPN can be separated into two possible components, we further split the analysis of SPN into an early and late component (mean amplitude time window for early SPN: 250–450 ms; late SPN: 450–600 ms). Considering that early and late SPN components show different topographies (for detailed Topographical ANalysis Of VAriance (TANOVA) and microstate analysis see Supplementary Materials – Appendix A), we analyzed the difference in topography for polarity-grouping and relative angle effects by using a group of electrodes around parietal-occipital sites (P7, P8, PO5, PO6, PO7, and PO8) compared to a central electrode group (C1, CZ, C2, CP1, CPZ, and CP2). We switched the sign of the parietal-occipital electrodes only, for individual participants, so that the statistical comparison between central and parietal-occipital locations would not be confounded by the switch in polarity of the ERP signal between parietal-occipital and central electrode locations. Note that we analyze a group of electrodes not in posterior locations, but over the center of the scalp, where there is a change in polarity of the signal compared to the back of the head. As such, the SPN calculated for the central group of electrodes is not a sustained posterior negativity per se, but a centrally distributed positivity. For clarity, we continue to use the terminology SPN to refer to the symmetry minus noise difference wave, irrespective of scalp distribution (central versus parietal-occipital).

Amplitude differences for P1, N1, and SPN components were assessed with repeated-measures ANalysis Of VAriance (ANOVA). For example, for PI/N1 analysis the factors were pattern type (symmetry versus noise), electrode (PO7 versus PO8), and either polarity (single versus polarity-grouped) or angle (0, 30, 60, 90 deg). When analyzing SPNs, the factors were the same except pattern type was not a factor (an SPN being the difference between symmetry and noise). Further analysis of the SPN included splitting the SPN time window into an early and late component (i.e., an additional factor of time window: early versus late), and analysis over two separate groups of six electrodes at two locations (i.e., including Location as a factor: parietal-occipital versus central). Therefore, SPN analysis resulted in four factors (SPN time window, polarity (or angle), location, and electrode). For all analyses, the Greenhouse–Geisser correction was applied, while post-hoc tests (paired-samples t-tests) were performed with a Bonferroni correction for multiple comparisons. We also show topographic differences corresponding to the difference wave, visualized using Cartool (Brunet et al., 2011). In addition, to confirm the presence of SPN, we carried out one-sample t-tests to examine whether SPN difference waves were significant from zero. The outcomes of one sample t-tests analysis are described in the Supplementary Materials – Appendix B.

## Results

### Behavioral results

**Figure 2.**
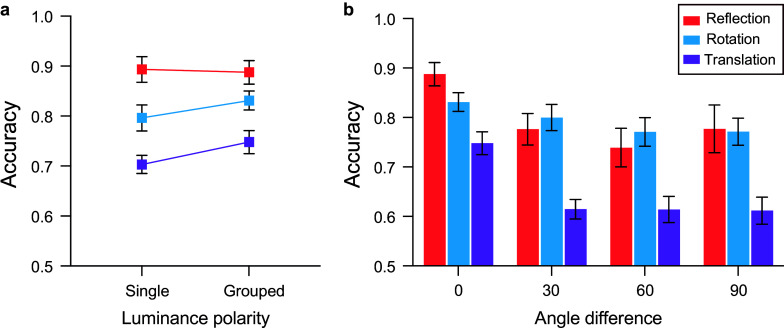
Accuracy (% correct responses) obtained with polarity-grouped patterns (a) with 0 deg angular difference between the symmetry and polarity axes, and single-polarity conditions; (b) a function of angular difference (0, 30, 60, 90 deg) between symmetry and polarity-grouping axes for reflection (red), rotation (blue), and translation (magenta) symmetric patterns. Error bars indicate the standard error of the mean (±1 SEM).

Figure 2 shows accuracy (% correct responses) obtained with single-polarity and polarity-grouped patterns as a function of angular difference between symmetry and polarity-grouping axes for reflection, rotation, and translation symmetric patterns. We first analyzed single-polarity versus 0 deg polarity-grouped conditions with aligned symmetry and luminance axes for all types of symmetries. We found that type of symmetry affected accuracy (F(1.749, 40.23) = 40.153, *p* < 0.0001, η_p_^2^ = 0.636), with accuracy highest for reflection symmetry, followed by rotation, and translation conditions (Figure 2a). All multiple comparisons between symmetry types were significant (all p’s < 0.015). Interestingly, the results indicate differences between single-polarity and 0 deg polarity-grouped conditions (F(1,23) = 7.59, *p* = 0.011, η_p_^2^ = 0.248), with higher accuracy for 0 deg polarity-grouped conditions. Further, a weak interaction effect between the type of symmetry and polarity (single versus 0 deg), (F(1.722, 39.614) = 3.038, p = 0.066, η_p_^2^ = 0.117), suggests that when polarity-grouped conditions were present, accuracy improved for rotation (t(23) = 2.648, p = 0.014, 95% CI [−0.062–0.008]) and translation symmetry (t(23) = 2.426, p = 0.024, 95% CI [−0.083–0.007]), but not for reflection symmetry (p = 0.683; see Figure 2a).

As for the other angular differences, Figure 2b indicates higher accuracy for 0 deg angular difference compared to all other angular difference conditions, for all types of symmetry. A two-way repeated-measures ANOVA with factors symmetry type (reflection versus rotation versus translation) and angular difference (0, 30, 60, 90) revealed a significant main effect of angular difference (F(1.664, 38.278) = 20.86, p < 0.0001, η_p_^2^ = 0.476), symmetry type (F(1.874, 43.09) = 20.43, p < 0.0001, η_p_^2^ = 0.470), and a significant interaction effect between symmetry and angular difference (F(3.046, 70.051) = 3.148, p = 0.03, η_p_^2^ = 0.120). Bonferroni-corrected multiple comparison tests showed that pairwise comparisons between 0 deg and all other angular differences (30, 60, 90) were significant for reflection, rotation, and translation symmetry conditions (all p’s < 0.0002), except for 0 versus 90 deg reflection (p = 0.132) and 0 versus 30 deg rotation (p = 0.243) symmetry. No other significant differences were found between angular conditions greater than zero (all p’s > 0.107). Overall, mean accuracy for reflection and rotation symmetry was comparable (~80%) and higher than translation symmetry (~65%).

### P1 & N1 analysis

**Figure 3.**
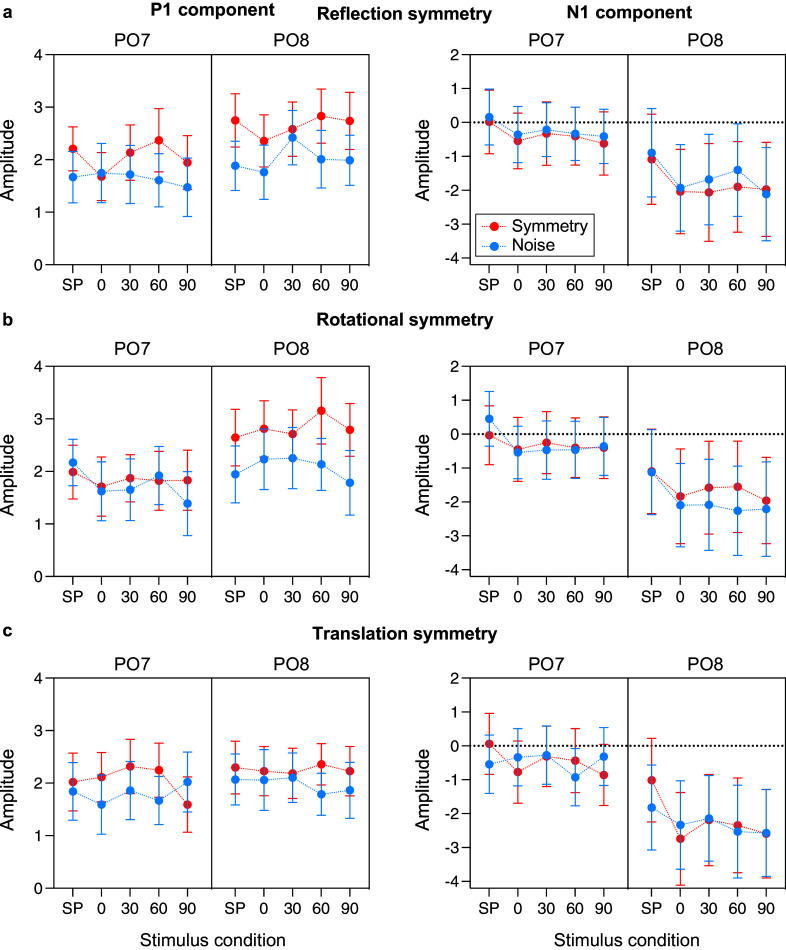
Mean amplitude of P1 (left panel) and N1 (right panel) waves respectively, measured at electrodes PO7 & PO8, for each symmetry type and all conditions, with the symmetric patterns shown by red symbols and noise patterns indicated by blue symbols. SP denotes the single-polarity condition (i.e., symmetric and noise patterns made of either all black or all white elements). The 0–90 conditions refer to the relative angle difference (in degrees) between luminance polarity and symmetry axes. Error bars indicate the standard error of the mean (±1 SEM). Note the separation between symmetry and noise in P1 mean amplitude in comparison to the N1 (compare red and blue symbols).

Figure 3 shows the mean amplitude of P1 (left panel) and N1 (right panel) waves respectively, measured at electrodes PO7 & PO8, for each symmetry type and all stimulus conditions, with the symmetric patterns shown by red symbols and noise patterns indicated by blue symbols.

The effect of luminance polarity was analyzed with three-way repeated-measures ANOVA, with factors of pattern type (symmetry versus noise), polarity (single versus polarity-grouped), and electrode (PO7 versus PO8). Similarly, to examine if the relative angle between symmetry and polarity-grouping axes affects the ERP response to symmetric and polarity-grouped patterns, we used a three-way repeated-measures ANOVA with factors pattern type (symmetry versus noise), angle (0, 30, 60, 90), and electrode (PO7 versus PO8). All models were conducted on each ERP component separately (P1, N1, and SPN).

For ***P1 amplitude***, reflection-symmetric conditions elicited larger amplitudes than noise (F(1,23) = 6.419, p < 0.019, η_p_^2^ = 0.218), and a larger amplitude for single compared to polarity-grouped (0 deg), (F(1,23) = 4.738, p = 0.04, η_p_^2^ = 0.171), but no significant interaction between symmetry and polarity was found (F(1,23) = 1.385, p = 0.251, η_p_^2^ = 0.057). When examining angular differences, we found again an increased P1 amplitude for symmetry compared to noise conditions (F(1,23) = 12.629, p = 0.002, η_p_^2^ = 0.354), but no other main effects or interactions were significant (all p’s > 0.169).

Rotational symmetry elicited larger P1 amplitudes than noise, but only at electrode PO8 (F(1, 23) = 5.617, p = 0.027, η_p_^2^ = 0.196). Similarly, we found an effect of polarity such that 0 deg polarity-grouped produced larger P1 amplitudes than single-polarity conditions, in the right hemisphere only (F(1, 23) = 8.849, p = 0.007, η_p_^2^ = 0.278). When examining angular differences, we found a significant effect of symmetry (F(1,23) = 6.469, p = 0.002, η_p_^2^ = 0.220), and an interaction effect highlighting that the P1 response to symmetry was largest at electrode PO8 (F(1,23) = 10.133, p = 0.004, η_p_^2^ = 0.306).

Translational symmetry elicited no differences in P1 mean amplitudes between single-polarity and polarity-grouped symmetric (0 deg) conditions (all p’s > 0.117). Similarly, when analyzing the effect of angle, we found a significant effect symmetry (F(1,23) = 4.991, p = 0.035, η_p_^2^ = 0.178), but no other notable differences in P1 mean amplitude (all p’s > 0.085). Overall, across all symmetry types, it appears that P1 amplitude is increased by symmetry in relation to noise but is insensitive to polarity changes or relative angular differences.

For ***N1 amplitude***, we analyzed single versus polarity-grouped (0 deg) conditions for reflection symmetry and found only a main effect of polarity (F(1,23) = 10.006, p = 0.004, η_p_^2^ = 0.303), such that the polarity-grouped conditions produced a larger N1 amplitude than single-polarity conditions. No other effects were found (all p’s > 0.074). Further, the N1 did not respond to angular differences between reflection symmetry and polarity axes, but there was weak evidence for a larger N1 in PO8 compared to PO7 (F(1,23) = 4.255, p = 0.051, η_p_^2^ = 0.156), with no other effects approaching significance in this model (all p’s > 0.238).

Like reflection, we found a polarity difference for rotation symmetry, such that N1 amplitude was largest for 0 deg polarity-grouped conditions compared to single-polarity conditions (F(1, 23) = 12.639, p = 0.002, η_p_^2^ = 0.355). All other effects were not significant (all p’s > 0.063). Analyzing the angular differences for rotation, no effects approached significance (all p’s > 0.093).

For translational symmetry, we found a polarity difference indicating larger N1 amplitudes for 0 deg polarity-grouped compared to single-polarity conditions (F(1, 23) = 9.446, p = 0.005, η_p_^2^ = 0.291). Further, we found an effect of symmetry, with noise conditions producing larger N1 amplitudes than translation (F(1,23) = 11.317, p = 0.003, η_p_^2^ = 0.330), which was largest in PO8 (F(1, 23) = 4.95, p = 0.036, η_p_^2^ = 0.177). Finally, a three-way interaction between symmetry, polarity, and electrode revealed that the larger N1 amplitudes for noise were driven by electrode PO8, with PO7 showing an opposite, but smaller, direction of effect (F(1,23) = 12.419, p = 0.002, η_p_^2^ = 0.351). The polarity difference was consistent across channels and symmetry type. When we analyzed the factor of angle, we found only an effect of electrode (F(1,23) = 7.488, p = 0.017, η_p_^2^ = 0.246), with N1 being the largest in the right hemisphere compared to the left. No other effects were significant (all p’s > 0.097).

In sum, we found that N1 amplitude is insensitive to symmetry or relative angular differences but is modulated by changes in polarity.

### Polarity effects on noise only conditions over P1, N1, and SPN

Given the N1 sensitivity to polarity for each type of symmetry, we examined if noise only conditions associated with the symmetry type were sensitive to polarity. Note that because of the 2IFC design each symmetry type was presented with its own noise condition, but noise stimuli across symmetry types were generated in the same manner. We therefore ran analyses separated by each symmetry type, for noise only conditions. We found that N1 was largest for polarity-grouped noise compared to single-polarity noise: for reflection symmetry trials, (F(1,23) = 7.53, p = 0.012, η_p_^2^ = 0.247); rotation (F(1,23) = 16.008, p < 0.001, η_p_^2^ = 0.410); translation trials – a polarity by electrode interaction (F(1,23) = 6.464, p = 0.018, η_p_^2^ = 0.185). There was no polarity sensitivity for P1 noise only conditions (p > 0.453).

**Figure 4.**
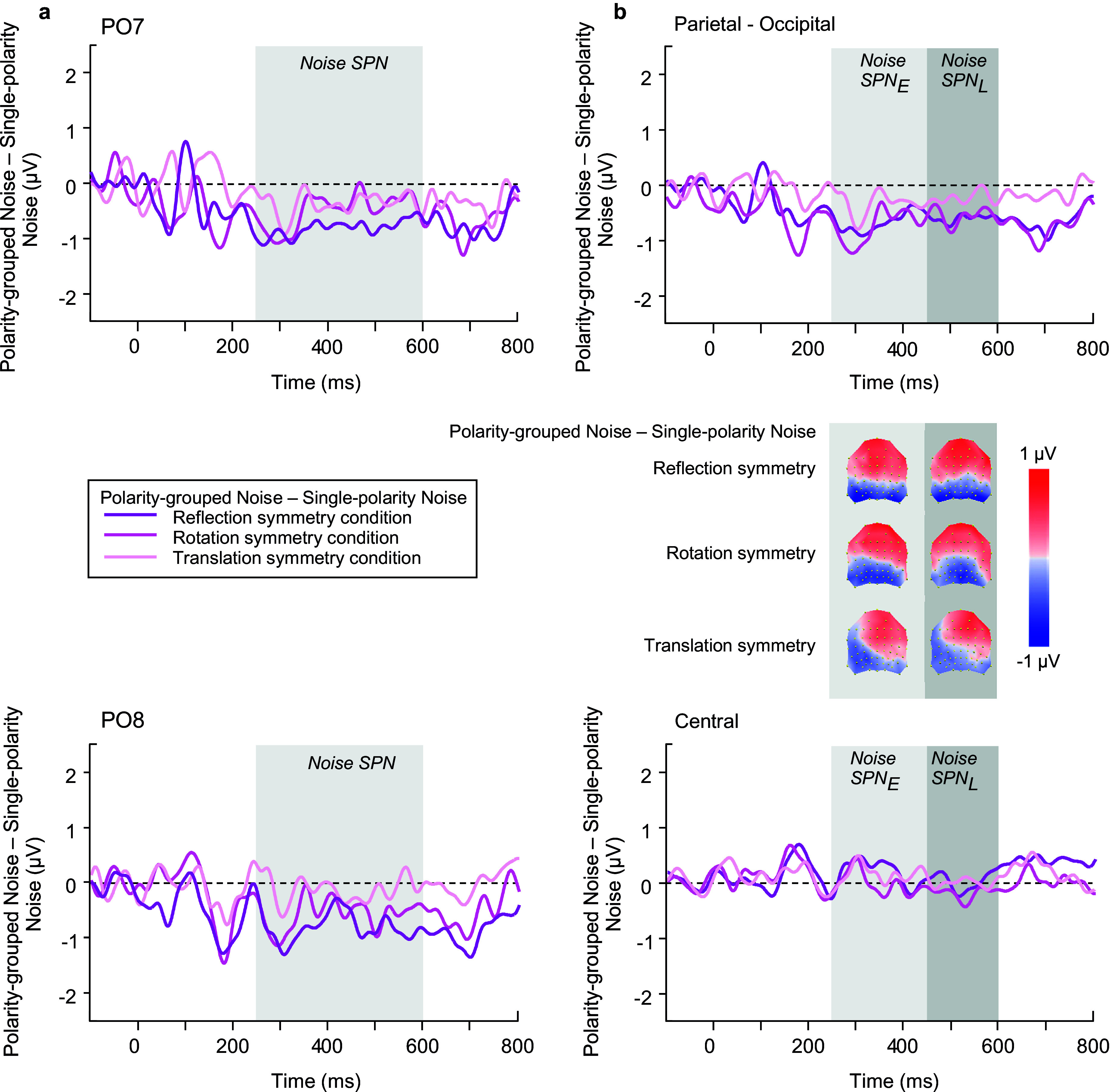
Waveforms and topographies depict the difference between polarity-grouped minus single-polarity noise conditions used in the 2IFC. This noise data are split by each pattern’s corresponding symmetry type (reflection, rotation, translation), resulting in a noise-SPN difference at (a) PO7 and PO8 electrodes; and (b) group of 6 electrodes at parietal-occipital (P7, PO7, PO5, P8, PO8, PO6), and central (C1, CZ, C2, CP1, CPZ, CP2) locations.

Before analyzing SPN amplitude for symmetry effects, we first evaluated whether there was a ***polarity-grouping effect in the noise conditions only***, over the SPN time window 250–600 ms, during the *symmetry detection task* (Figure 4a). We found, perhaps surprisingly, that noise types associated with the type of symmetry task (reflection, rotation, translation) were affected differentially by polarity-grouping (single versus polarity-grouped), such that there were large differences between reflection-noise conditions, marginal differences between rotation-noise conditions, and no differences between translation-noise (compare light, intermediate, and dark purple lines in Figure 4a); for reflection: t(23) = −3.137, p = 0.005, d = −0.640 for PO7; t(23) = −3.278, p = 0.003, d = −0.669 for PO8; for rotation: t(23) = −1.79, p = 0.087, d = −0.365 for PO7; t(23) = −1.84, p = 0.079, d = −0.376 for PO8; for translation: t(23) = −1.535, p = 0.138, d = −0.313 for PO7; t(23) = −0.386, p = 0.703, d = −0.079 for PO8. We suggest that these differences may reflect the ease of the symmetry detection task.

### SPN analysis at electrodes PO7 and PO8

**Figure 5.**
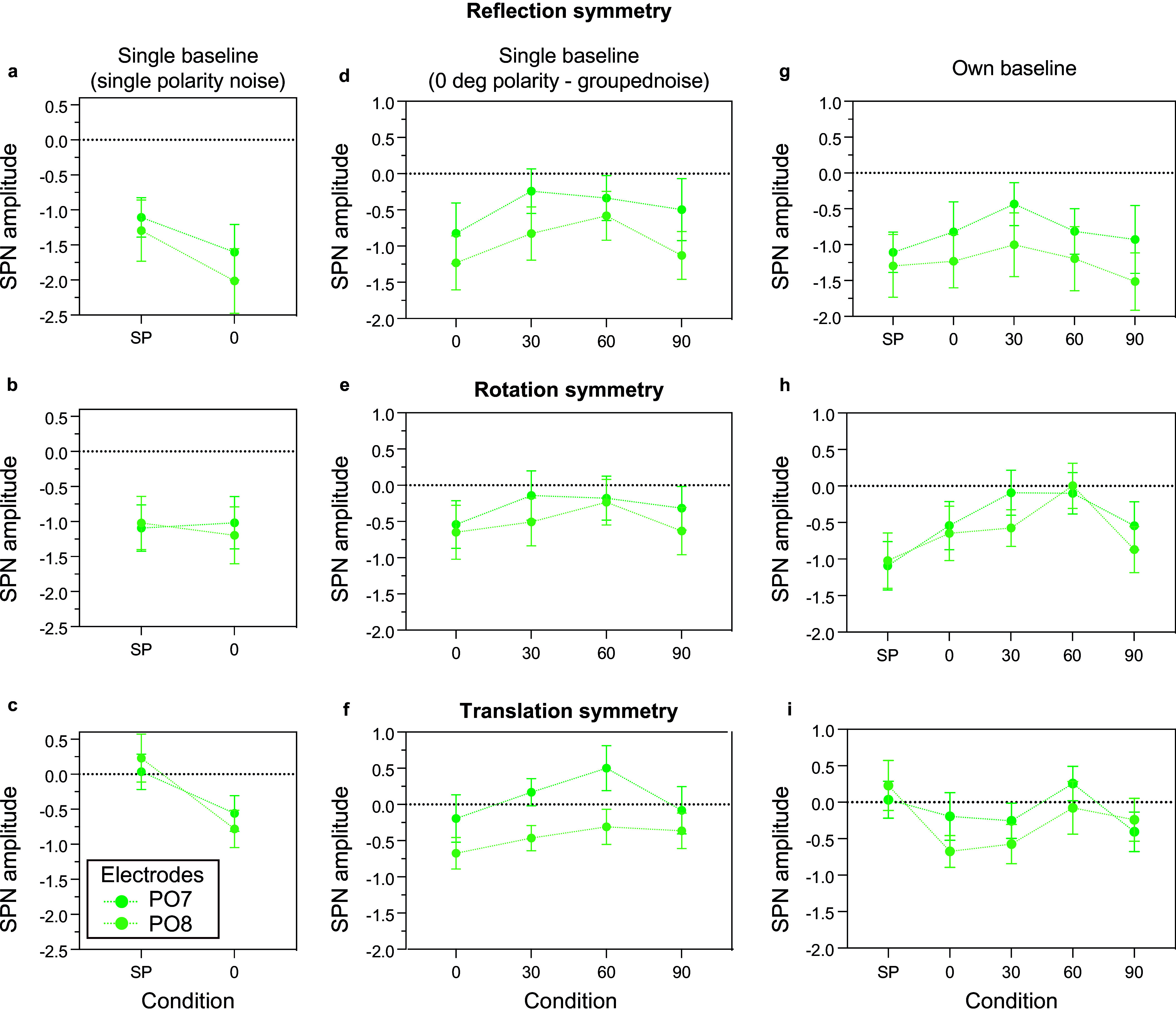
SPN mean amplitude at PO7 (dark green) and PO8 (light green) electrode locations for reflection (top panels), rotation (middle panels), and translation (bottom panels) symmetry. The SPN amplitude was calculated as the difference between symmetry and (a–c) a single baseline (single-polarity noise) condition; (d–f) single baseline (0 deg polarity-grouped noise) condition for each relative angle (0, 30, 60, 90) condition; (g–i) the own corresponding noise condition.

Since differences between noise conditions are present for single and 0 deg polarity-grouped conditions, we next analyzed ***SPN difference waves*** between symmetry conditions (single-polarity, 0 deg polarity-grouped) with respect to one baseline (single-polarity noise) for each symmetry type (reflection, rotation, translation) at PO7 and PO8 electrode (light and dark green lines in Figure 5a–c).

We examined the effects of polarity-grouping and relative angle independently, by using two-way repeated-measures ANOVAs with factors of polarity (polarity-grouped versus single-polarity) or relative angle (0, 30, 60, 90) and electrode (PO7 versus PO8), for the three symmetry types separately. We found an effect of polarity on translation-symmetry (F(1,23) = 11.108, p = 0.003, η_p_^2^ = 0.326) with increased amplitude for polarity-grouped compared to single-polarity condition, a weak effect for reflection symmetry (F(1,23) = 3.442, p = 0.076, η_p_^2^ = 0.130), and no effect of polarity-grouping for rotation (p > 0.843). When examining relative angle (Figure 5d–f), we found no effect on SPN for reflection, rotation, and translation symmetry (all p’s > 0.146), and no effect of electrode location (PO7 versus PO8; all p’s > 0.129) except for translation symmetry (F(1,23) = 7.372, p = 0.012, η_p_^2^ = 0.243; see Figure 5f). When examining one-sample t-tests, all SPN difference waves were significantly different from zero except for translation single-polarity SPN (see Supplementary Materials – Appendix B).

Similar analysis of SPN differences was carried out by considering each symmetry type with their corresponding noise pattern (own baseline, Figure 5g–i) instead of a single baseline (Figure 5a–f). The analysis of SPN mean amplitude *between 250 and 600 ms* showed that for single-polarity versus 0 deg polarity-grouped patterns (p = 0.619 for reflection; p = 0.201 for rotation), and for differences between angles (0, 30, 60, 90; p = 0.615 for reflection; p = 0.129 for rotation), there were no significant effects between conditions for both reflection- and rotational-symmetric patterns. For translation, there was a significant interaction between polarity and electrode site (F(1,23) = 4.47, p = 0.046, η_p_^2^ = 0.163), which appears to be an anomaly caused by the lack of SPN differences from zero for single-polarity in comparison to the 0 deg polarity-grouped condition at PO8 (see Figure 5i and Supplementary materials – Appendix B). As for the effect of angle, none of the effects were found significant (p > 0.175). These results suggest that SPN differences for all symmetry types were not modulated by luminance polarity or changes in polarity and symmetry axes.

### Noise analysis between early and late SPN time window

Based upon our microstate segmentation analysis (see Supplementary Materials – Appendix A) and our previous findings (Wright et al., 2018), we further split the analysis of SPN data into early (250–450 ms) and late (450–600 ms) time windows using a group of electrodes around parietal-occipital (P7, P8, PO5, PO6, PO7, and PO8) and central (C1, CZ, C2, CP1, CPZ, and CP2) electrode sites. For each of these analyses, we also tested whether polarity-grouped minus single-polarity reflection noise conditions were different from zero at central and parietal-occipital locations (see Supplementary Materials – Appendix B).

**Figure 6.**
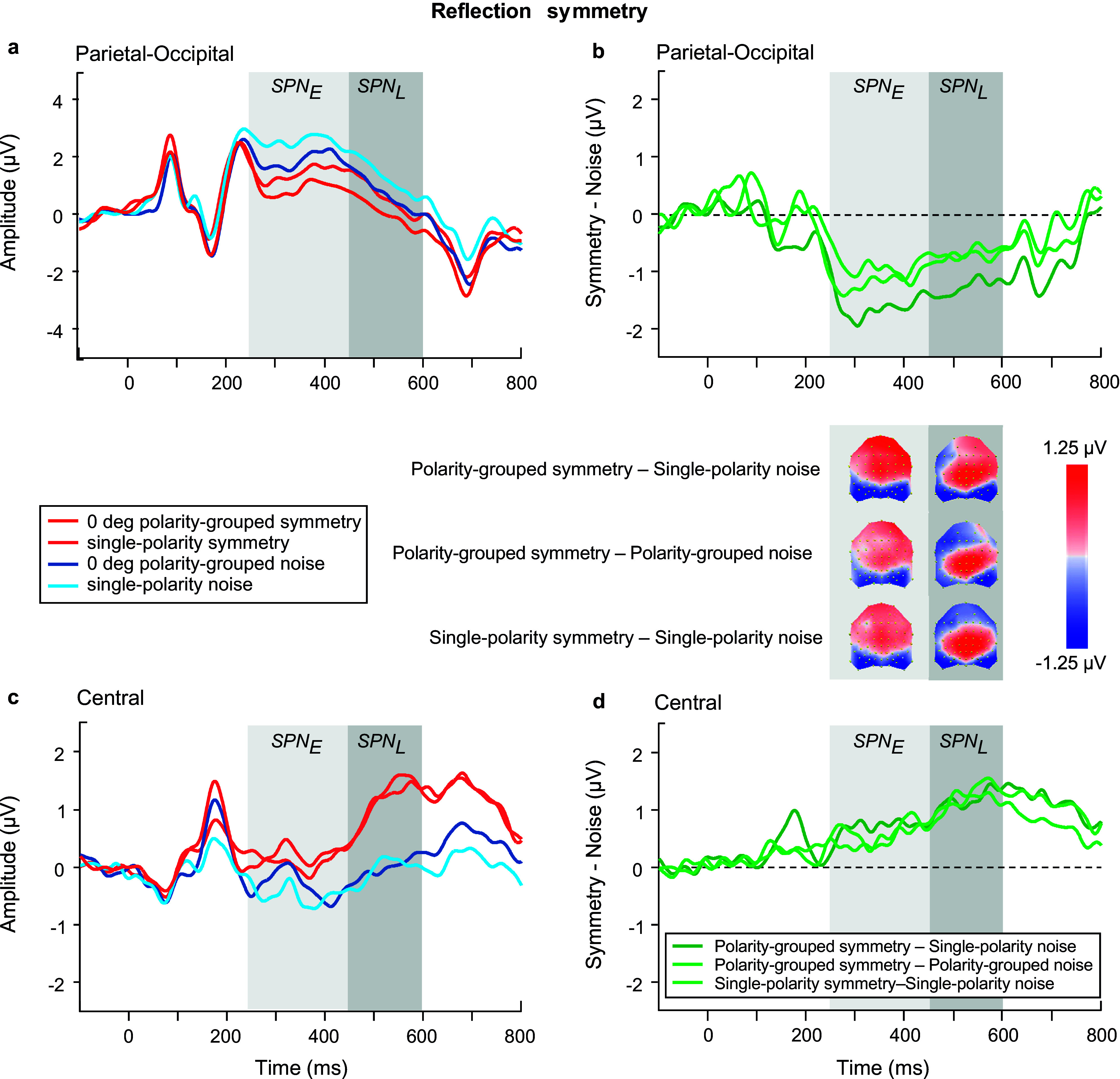
Reflection symmetry results. (a,c) Grand-average ERPs for single-polarity and 0 deg polarity-grouped symmetric (light and dark red) and noise (light and dark blue) patterns measured over (a) parietal-occipital and (c) central electrodes. Waveforms depict the average of 6 electrodes (P7, P8, PO5, PO6, PO7, PO8) at parietal-occipital locations (a) and 6 electrodes (C1, CZ, C2, CP1, CPZ, CP2) at central locations (c) respectively. (b,d) Difference waves (symmetry minus noise) for three conditions: single-polarity symmetry minus single-polarity noise (light green), 0 deg polarity-grouped symmetry minus single-polarity noise (dark green), 0 deg polarity-grouped symmetry minus polarity-grouped noise (intermediate green) over parietal-occipital (b) and central (d) electrodes. Topographic difference maps corresponding to early (250–450 ms) and late (450–600 ms) SPN time window for each of the three conditions are shown below (b).

We began with analyzing the ERPs for *polarity grouped noise conditions only* in the SPN time windows (see blue waveforms in Figure 6a,c), using an ANOVA model with factors of time window, polarity, location, and electrode. Polarity-grouped noise associated with reflection-symmetry (i.e., reflection-noise) elicited lower mean ERP amplitudes than single-polarity noise (F(1,23) = 10.671, p = 0.003, η_p_^2^ = 0.317). No differences were found for rotation (Figure 8a,c) or translation-noise (Figure 10a,c) (rotation: F(1,23) = 0.823, p = 0.374, η_p_^2^ = 0.035; translation: F(1,23) = 1.582, p = 0.221, η_p_^2^ = 0.064). There was a main effect of time window, with larger ERP amplitudes observed in the early time window compared to the late, for all symmetry types (reflection: F(1,23) = 23.69, p < 0.001, η_p_^2^ = 0.507; rotation: F(1,23) = 23.083, p < 0.001, η_p_^2^ = 0.501; translation: F(1,23) = 15.494, p < 0.001, η_p_^2^ = 0.402; see blue waveforms in Figure 6a,c; Figure 8a,c and Figure 10a,c). However, no time window x polarity interaction was found for any symmetry type (all p’s > 0.076), demonstrating that the differences between polarity-grouped and single-polarity noise conditions do not change over time.

**Figure 7.**
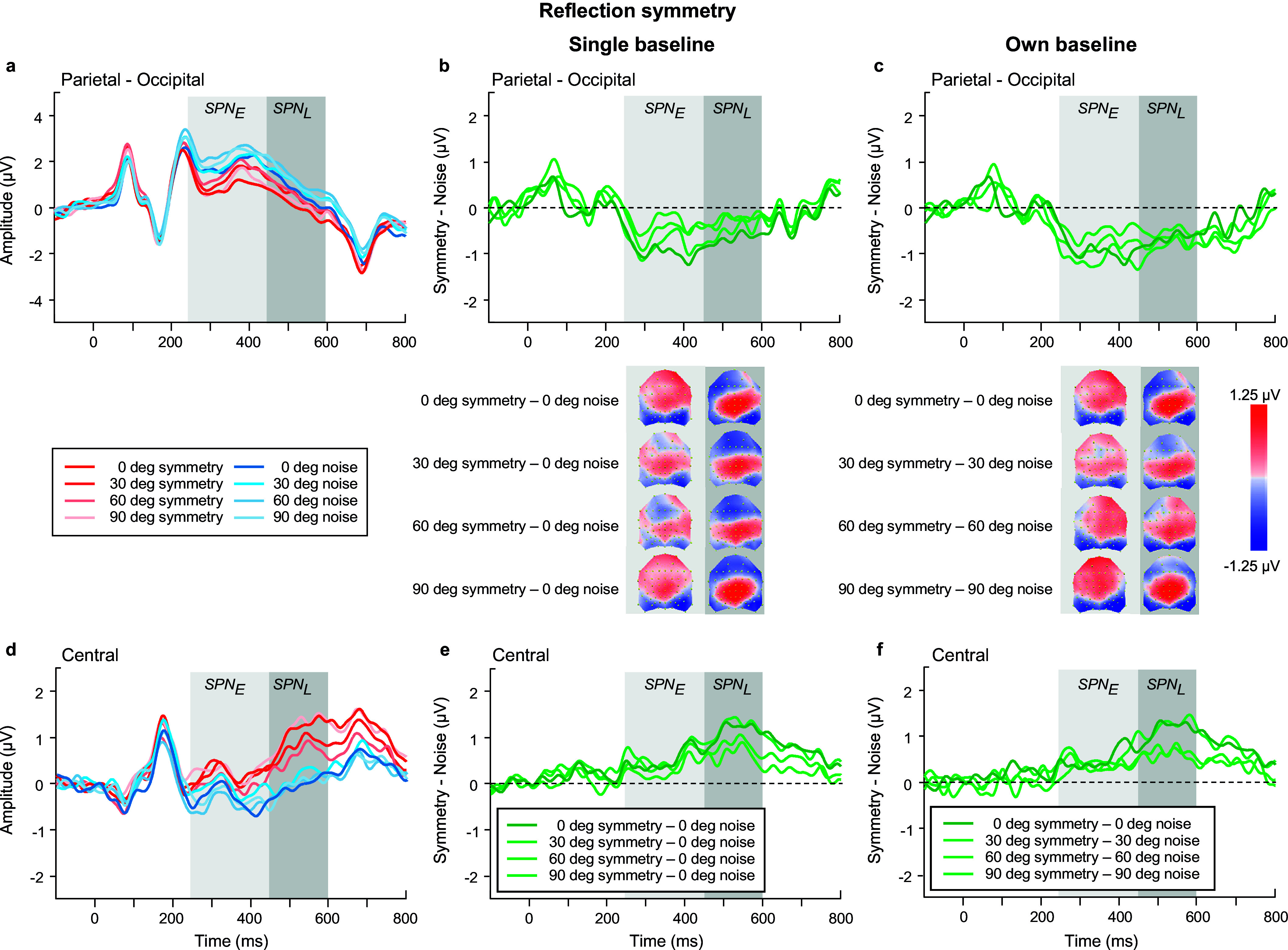
Reflection symmetry results. (a,d) Grand-average ERPs for noise (blue) and reflection-symmetric (red) patterns with an angular difference between symmetric and polarity-grouping axes of 0 (axes coincide; dark colors), 30, 60, and 90 deg (orthogonal axes; light colors) measured over (a) parietal-occipital and (d) central electrodes. Waveforms depict the average of 6 electrodes (P7, P8, PO5, PO6, PO7, PO8) at parietal-occipital locations (a) and 6 electrodes (C1, CZ, C2, CP1, CPZ, CP2) at central locations (d) respectively. (b,e) SPN difference waves (symmetry minus 0 deg noise) for four angle conditions: 0, 30, 60, 90 deg (dark to light green) over parietal-occipital (b) and central (e) electrodes were calculated in respect to the same baseline condition of 0 deg noise. (c,f) SPN difference waves (symmetry minus corresponding noise) for four angle conditions: 0, 30, 60, 90 deg (dark to light green) over parietal-occipital (c) and central (f) electrodes. Topographic difference maps corresponding to early (250–450 ms) and late (450–600 ms) SPN time window and for each angle condition are shown below (b) and (c).

Further, we analyzed just the noise conditions for each symmetry type when the luminance polarity axis was angled away from vertical (0, 30, 60, 90 deg conditions) – see blue waveforms in Figure 7a,c; Figure9a,c and Figure11a,c). For noise associated with each type of symmetry, ERP amplitude was largest in the early SPN time window (reflection: F(1,23) = 18.995, p < 0.001, η_p_^2^ = 0.452; rotation: F(1,23) = 20.151, p < 0.001, η_p_^2^ = 0.467; translation: F(1,23) = 14.496, p < 0.001, η_p_^2^ = 0.387). No effect of luminance polarity angle was found (reflection: F(1,23) = 1.935, p = 0.152, η_p_^2^ = 0.078; rotation: F(1,23) = 2.104, p = 0.113, η_p_^2^ = 0.084; translation: F(1,23) = 0.336, p = 0.76, η_p_^2^ = 0.014), suggesting that the angle of the luminance polarity axis had no significant effect on the perception of noise. We also tested whether noise-SPN corresponding to angular differences (30, 60, 90 deg) minus 0 deg polarity conditions were different from zero at central and parietal-occipital locations; these noise-SPN differences were not significant from zero (all p’s > 0.06 for reflection; all p’s > 0.133 for rotation; all p’s > 0.288 for translation) indicating that luminance polarity angle changes do not induce SPN-like effects by themselves.

### Early versus Late SPN in relation to a single noise baseline

**Figure 8.**
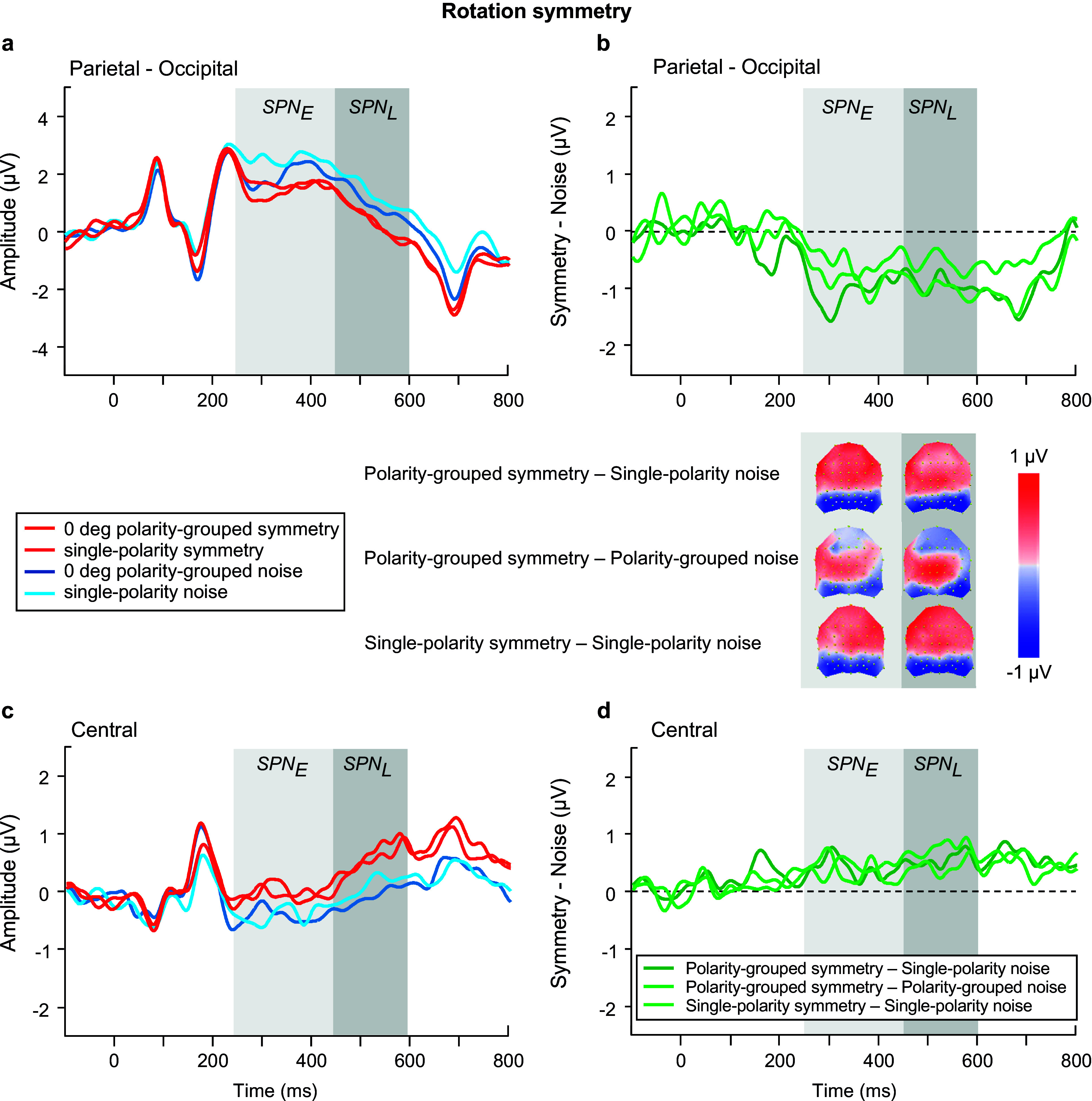
Rotation symmetry results. (a,c) Grand-average ERPs for single-polarity and 0 deg polarity-grouped symmetric (light and dark red) and noise (light and dark blue) patterns measured over (a) parietal-occipital and (c) central electrodes. Waveforms depict the average of 6 electrodes (P7, P8, PO5, PO6, PO7, PO8) at parietal-occipital locations (a) and 6 electrodes (C1, CZ, C2, CP1, CPZ, CP2) at central locations (c) respectively. (b,d) Difference waves (symmetry minus noise) for three conditions: single-polarity symmetry minus single-polarity noise (light green), 0 deg polarity-grouped symmetry minus single-polarity noise (dark green), 0 deg polarity-grouped symmetry minus polarity-grouped noise (intermediate green) over parietal-occipital (b) and central (d) electrodes. Topographic difference maps corresponding to early (250–450 ms) and late (450–600 ms) SPN time window for each of the three conditions are shown below (b).

We next analyzed the SPN differences between single-polarity and 0 deg polarity-grouped conditions with respect to the single-polarity noise condition for each type of symmetry (Figures 6, 8, 10). We use a four-way repeated-measure ANOVA with factor time window (early versus late), polarity (single versus polarity-grouped), location (central versus parietal-occipital), and electrode (six electrodes). For reflection symmetry (Figure 6), an interaction between polarity and location showed that the difference between polarity SPNs was largest at parietal-occipital electrode locations only (F(1,23) = 6.633, p = 0.017, η_p_^2^ = 0.224; compare light and dark green in Figure 6b). The interaction between time window and location showed that SPN amplitude was largest in the early time window at parietal-occipital electrode locations, and largest at central electrode locations in the late time window (F(1,23) = 34.524, p < 0.001, η_p_^2^ = 0.6; compare Figure 6b & 6d).

For rotation symmetry (Figure 8), SPN amplitude was larger in the parietal-occipital compared to central locations (F(1,23) = 4.39, p = 0.048, η_p_^2^ = 0.166; Figure 8b,d). Further, a three-way interaction between time window, polarity, and location, showed that the 0 deg polarity-grouped condition elicited a larger SPN amplitude than single-polarity only in the early time window for the parietal-occipital location, and this difference disappeared in the later time window (F(1,23) = 9.383, p = 0.006, η_p_^2^ = 0.299; see Figure 8b).

For translation (Figure 10), we found that the polarity-grouped condition elicited a larger SPN compared to single-polarity (F(1,23) = 9.227, p = 0.006, η_p_^2^ = 0.295), and these differences appeared to be larger at the parietal compared to central locations (F(1,23) = 4.034, p = 0.057, η_p_^2^ = 0.155; see Figure 10b,d). In addition, for both polarity-grouping and relative angle difference analyses, we also tested whether SPN magnitude was different from zero at central and parietal-occipital locations and early and late time windows (see Supplementary Materials – Appendix B).

As for the **relative angular difference** effects on the SPN for reflection symmetry (Figure 7), we found a main effect of angle (F(2.25,51.65) = 3.138, p = 0.046, η_p_^2^ = 0.12), with larger amplitude for relative angular differences of 0 and 90 deg compared to other angles (30, 60 deg). A time window by location interaction (F(1,23) = 26.806, p < 0.001, η_p_^2^ = 0.538) displayed larger SPN amplitudes in the early time window over the parietal-occipital electrodes, but greater amplitudes in the late time window over central locations (compare Figure 7b,e). A three-way interaction between time window, angle, and location (F(2.79,64.2) = 4.757, p = 0.006, η_p_^2^ = 0.171) suggests that the changes in SPN amplitude due to angular differences were greater in the early time window over parietal-occipital electrodes and in the late time window over central electrodes.

**Figure 9.**
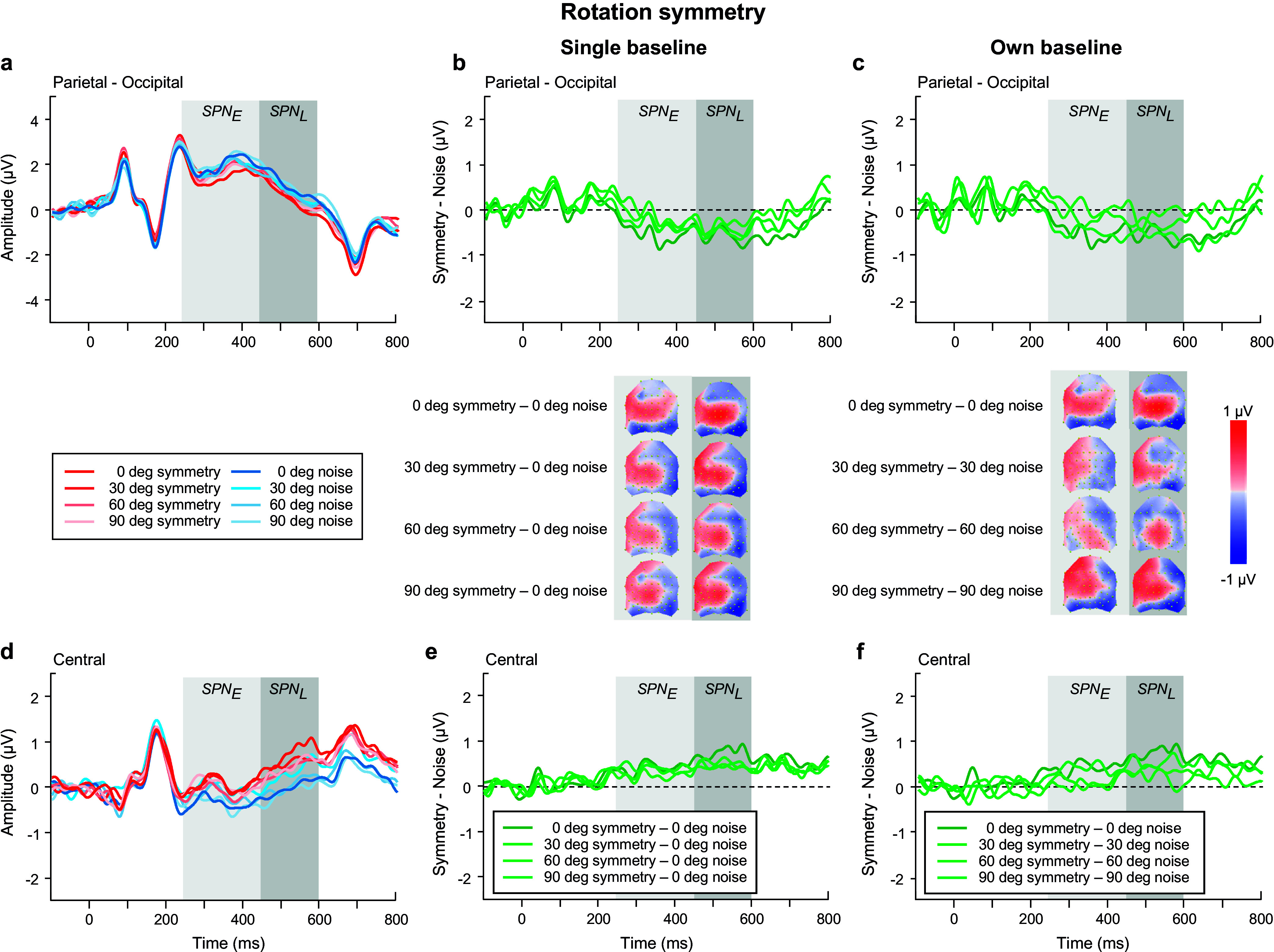
Rotation symmetry results. (a,d) Grand-average ERPs for noise (blue) and reflection-symmetric (red) patterns with an angular difference between symmetric and polarity-grouping axes of 0 (axes coincide; dark colors), 30, 60, and 90 deg (orthogonal axes; light colors) measured over (a) parietal-occipital and (d) central electrodes. Waveforms depict the average of 6 electrodes (P7, P8, PO5, PO6, PO7, PO8) at parietal-occipital locations (a) and 6 electrodes (C1, CZ, C2, CP1, CPZ, CP2) at central locations (d) respectively. (b,e) SPN difference waves (symmetry minus 0 deg noise) for four angle conditions: 0, 30, 60, 90 deg (dark to light green) over parietal-occipital (b) and central (e) electrodes were calculated in respect to the same baseline condition of 0 deg noise. (c,f) SPN difference waves (symmetry minus corresponding noise) for four angle conditions: 0, 30, 60, 90 deg (dark to light green) over parietal-occipital (c) and central (f) electrodes. Topographic difference maps corresponding to early (250–450 ms) and late (450–600 ms) SPN time window and for each angle condition are shown below (b) and (c).

For the rotation symmetry (Figure 9), we found only a three-way interaction between time window, angle, and location (F(3,69) = 3.652, p = 0.03, η_p_^2^ = 0.137) suggesting larger SPN amplitudes over the late time window in both central and parietal-occipital locations, with 0 deg amplitude larger compared to all other angle conditions (compare Figure 9b,e).

For translation symmetry (Figure 11), we found a main effect of time window (F(1,23) = 4.3, p = 0.05, η_p_^2^ = 0.157) and an interaction between time window and location (F(1,23) = 4.811, p = 0.039, η_p_^2^ = 0.173), suggesting larger SPN amplitudes over the late time window at the central locations (Figure 11e). In addition, a significant relative angle difference was found (F(3,69) = 3.613, p = 0.023, η_p_^2^ = 0.136) with smaller SPN amplitude for 60 deg compared to all other angle (0, 30, 90 deg) conditions (0 versus 60, p = 0.004; 30 versus 60, p = 0.077; 90 versus 60, p = 0.056).

### Early versus Late SPN in relation to corresponding noise baselines

The same SPN analysis was conducted but with respect to each condition’s own noise baseline, separately for each type of symmetry.

**Figure 10.**
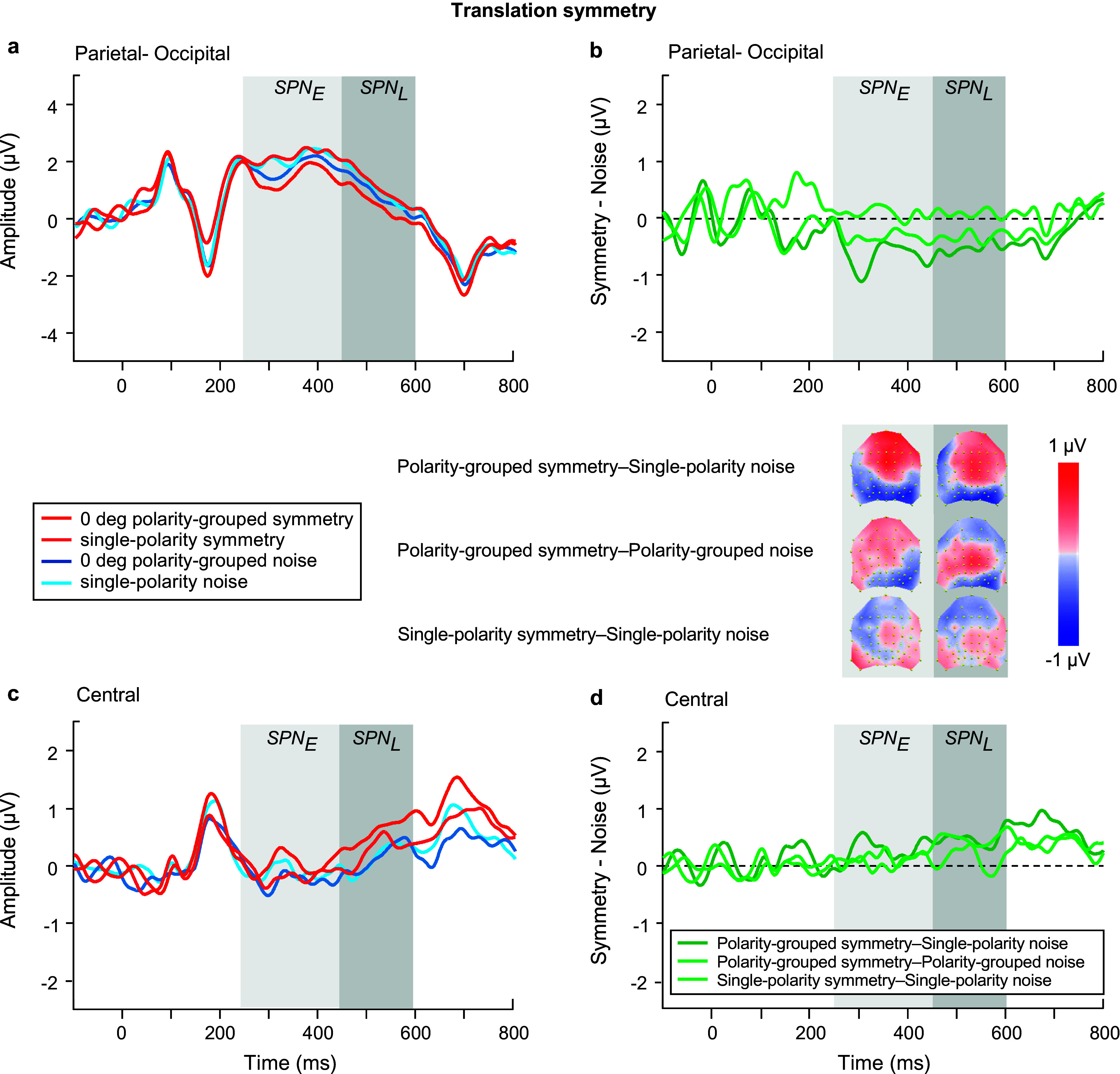
Translation symmetry results. (a,c) Grand-average ERPs for single-polarity and 0 deg polarity-grouped symmetric (light and dark red) and noise (light and dark blue) patterns measured over (a) parietal-occipital and (c) central electrodes. Waveforms depict the average of 6 electrodes (P7, P8, PO5, PO6, PO7, PO8) at parietal-occipital locations (a) and 6 electrodes (C1, CZ, C2, CP1, CPZ, CP2) at central locations (c) respectively. (b,d) Difference waves (symmetry minus noise) for three conditions: single-polarity symmetry minus single-polarity noise (light green), 0 deg polarity-grouped symmetry minus single-polarity noise (dark green), 0 deg polarity-grouped symmetry minus polarity-grouped noise (intermediate green) over parietal-occipital (b) and central (d) electrodes. Topographic difference maps corresponding to early (250–450 ms) and late (450–600 ms) SPN time window for each of the three conditions are shown below (b).

First, we examined the effect of ***luminance polarity*** (Figures 6, 8, 10). For *reflection symmetry* (Figure 6b,d), the analysis showed no overall effect of time window, polarity, or location (all p’s > 0.137). Critically, we found an interaction between time window and location (F(1,23) = 62.956, p < 0.0001, η_p_^2^ = 0.732), highlighting the differences between early and late SPN components due to scalp location, with early SPN being largest for parietal-occipital sites, and diminished for central electrodes, yet with the late SPN showing the opposite effect (increased at central and reduced at parietal-occipital electrode sites). For *rotation symmetry* (Figure 8), an analysis of the single-polarity versus 0 deg polarity-grouped condition showed no overall effect of polarity, location, or interaction effects (all p’s > 0.088). For *translation symmetry* (Figure 10), an analysis of the single-polarity versus 0 deg polarity-grouped conditions showed no overall main effects of time window, polarity, or location (all p’s > 0.085) but a significant three-way interaction between time window, polarity, and location (F(1,23) = 6.639, p = 0.017, η_p_^2^ = 0.224). To further understand this interaction, we separately examined central and parietal-occipital electrode locations, which showed no changes in SPN amplitudes for polarity or time window over parietal-occipital electrodes (all p’s > 0.119). Critically, central electrode sites were increased in amplitude at the late SPN time window compared to the early time window (F(1,23) = 7.691, p = 0.011, η_p_^2^ = 0.251), which was driven by a large increase in amplitude for the 0 deg polarity-grouped compared to the single-polarity condition (F(1,23) = 8.542, p = 0.008, η_p_^2^ = 0.271; see lighter green lines in Figure 10b,d).

In sum, when analyzing with respect to corresponding noise baselines, no main effect of polarity is observable for all types of symmetry (in contrast with the analysis to a single baseline, section “Early versus Late SPN in relation to a single noise baseline”).

Additionally, for both polarity-grouping and relative angle difference analyses, we also tested whether SPN magnitude was different from zero at central and parietal-occipital locations and early and late time windows (see Supplementary Materials – Appendix B).

When we analyzed the effect of ***relative angular difference*** (0, 30, 60, 90 deg) for *reflection symmetry* (Figure 7), we found no significant main effects of time window, angle, and location (all p’s > 0.125), but again we found a significant interaction of time window by location (F(1, 23) = 24.85, p < 0.001, η_p_^2^ = 0.519), highlighting the changes in early and late SPN time windows over different topographic locations (see Figure 7c topographies). Further, a three-way interaction between time window, angle, and location was significant (F(2.84, 65.36) = 5.466, p = 0.002, η_p_^2^ = 0.192) which was driven by the change from early to late SPN across central electrodes for 0 deg (t(23) = −4.904. p = 0.007) and 90 deg (t(23) = −4.709, p = 0.012) angular difference conditions (Figure 7f). This finding was confirmed when analyses were split by location, with no effects observed at parietal-occipital electrodes (all p’s > 0.233), but an interaction between cardinal (0, 90 deg) and non-cardinal (30, 60 deg) angles was found in the late time window at central electrodes (F(2.88, 66.2) = 3.765, p = 0.016, η_p_^2^ = 0.141). This suggests that cardinal (0, 90 deg) axes elicited an increase in SPN amplitude compared to 30 & 60 deg conditions, with these amplitude differences larger in the late time window over the center of the scalp than the parietal-occipital.

As for *rotation symmetry* (Figure 9), we found marginally significant effects of time window (F(1,23) = 4.281, p = 0.05, η_p_^2^ = 0.157) and angle (F(2.56, 58.85) = 2.666, p = 0.065, η_p_^2^ = 0.104). Further analysis of time window by location (central versus parietal-occipital) indicates an increased SPN amplitude for the late time window compared to the early window, at central electrode sites (F(1,23) = 11.647, p = 0.002, η_p_^2^ = 0.336) which was not present for parietal-occipital electrodes (p = 0.35; compare Figure 9c and 9f).

**Figure 11.**
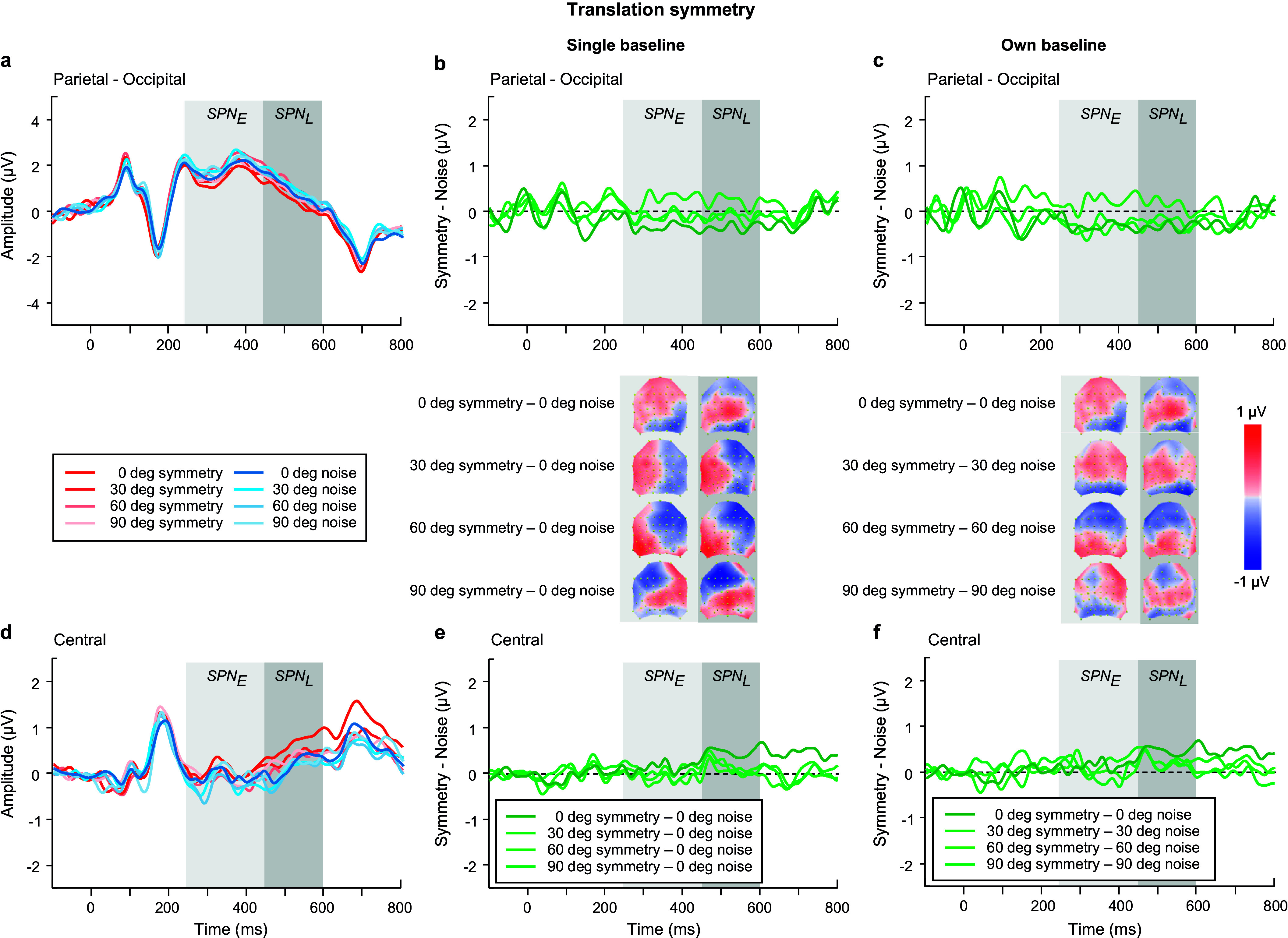
Translation symmetry results. (a,d) Grand-average ERPs for noise (blue) and reflection-symmetric (red) patterns with an angular difference between symmetric and polarity-grouping axes of 0 (axes coincide; dark colors), 30, 60, and 90 deg (orthogonal axes; light colors) measured over (a) parietal-occipital and (d) central electrodes. Waveforms depict the average of 6 electrodes (P7, P8, PO5, PO6, PO7, PO8) at parietal-occipital locations (a) and 6 electrodes (C1, CZ, C2, CP1, CPZ, CP2) at central locations (d) respectively. (b,e) SPN difference waves (symmetry minus 0 deg noise) for four angle conditions: 0, 30, 60, 90 deg (dark to light green) over parietal-occipital (b) and central (e) electrodes were calculated with respect to the same baseline condition of 0 deg noise. (c,f) SPN difference waves (symmetry minus corresponding noise) for four angle conditions: 0, 30, 60, 90 deg (dark to light green) over parietal-occipital (c) and central (f) electrodes. Topographic difference maps corresponding to early (250–450 ms) and late (450–600 ms) SPN time window and for each angle condition are shown below (b) and (c).

With regards to *translation symmetry* (Fig.11), our analysis of the angular differences showed a significant effect of SPN time window (F(1,23) = 9.557, p = 0.005, η_p_^2^ = 0.294) and an interaction between time window and location (F(1,23) = 10.317, p = 0.004, η_p_^2^ = 0.310), such that an increase in SPN amplitude for the late time window was observed only for central electrode sites (F(1,23) = 24.593, p < 0.001, η_p_^2^ = 0.517; compare Figure 11c and 11f).

Overall, we found effects of relative angle for reflection symmetry only (0, 90 deg versus 30, 60 deg) when corresponding noise baselines are used. This complements our analysis using a single baseline (section “Early versus Late SPN in relation to a single noise baseline”), which showed effects for reflection (0, 90 deg versus 30, 60 deg), rotation (0 deg versus others), and translation (60 deg versus others).

## Discussion

We aimed to uncover the neural mechanisms involved in the interaction between luminance polarity and symmetry by manipulating the angular differences between symmetry and luminance polarity axes. *Behavioral* data showed that accuracy for symmetry detection was higher for reflection, followed by rotation and then translation symmetry, and for 0 deg polarity-grouped than single-polarity conditions only for rotation and translation symmetry. Moreover, 0 deg conditions were also higher compared to all other angular differences conditions (except for rotation 0 versus 30 deg), irrespective of symmetry type. Interestingly, for 30, 60, 90 deg translation symmetry conditions, participants’ accuracy was notably reduced, with performance being only just above chance.

With regard to EEG, early visual components of the ERP signal, *P1* and *N1*, were sensitive to symmetry and grouping by luminance polarity, respectively, but critically, were unresponsive to changes in the relative angular differences between luminance polarity and symmetry axes. One important finding was the larger ERP amplitude for polarity-grouped 0 deg noise in comparison to single-polarity noise, evident in noise SPN differences. Noise SPN showed strong responses to reflection symmetry, weak for rotation, and absent for translation. These results suggest that luminance polarity grouping (Gestalts/grouping phenomena) also affects the typical SPN responses to symmetry. Although noise conditions differed in luminance polarity grouping (single versus polarity-grouped), we found similar polarity-grouping effects across the whole SPN time window for symmetry, at electrodes PO7 and PO8 only. That is, the SPN response to symmetry was larger for polarity-grouped compared to single-polarity conditions for translation and reflection (weak evidence for an effect) symmetry, irrespective of baseline.

Furthermore, across the SPN time window, there were clear changes in scalp topography, which indicated a shift in the focus of SPN activity from parietal-occipital locations in the early portion of the time window to centrally located channels in the later portion. Our analyses of early and late SPN time windows confirmed these changes in location, revealing that 0 and 90 deg conditions produced larger *late SPN* time window differences over the central locations compared to 30 and 60 deg relative angular differences for reflection symmetry. For rotation and translation symmetry, 0 deg polarity-grouped conditions consistently produced a larger SPN difference, again in the *late* SPN time window over central locations, compared to single-polarity conditions, but otherwise no effect of relative angular differences was found. The strongest effects found in the late SPN time window somewhat resemble the topographic distribution of the P300 component (Donchin and Coles, 1988). This would also be supported by recent work suggesting increased centrally distributed activity, linked to posterior cingulate cortex, during symmetry discrimination when symmetry is more salient and behaviorally relevant (Tyson-Carr et al., 2021).

In contrast, the *early* SPN time window displayed no sensitivity to luminance polarity or angular differences at either parietal-occipital or central scalp locations across all symmetry types, when analyzed with their own corresponding noise baselines. However, when evaluating SPN in respect to a single baseline, an effect of polarity was present over early and late time windows at parietal-occipital electrodes only.

Firstly, it is important to note that the majority of studies examining neural correlates of symmetry perception have predominantly analyzed the SPN difference wave only at PO7 and PO8 electrode locations (e.g., Palumbo et al., 2015), while less emphasis was put on the analysis of P1 and N1 components. Here, our analyses showed that the P1 is to some degree sensitive to symmetry, with an enhanced P1 amplitude at PO7 and PO8 locations for reflection symmetry, and at PO8 only for rotation and translation symmetry. In contrast, the N1 wave displayed no sensitivity to any symmetry type but was right-lateralized and responsive to luminance polarity changes. We do know that the N1 is consistently right-lateralized in other perceptual domains, e.g., perception of faces which contain a vertical axis of reflection symmetry (Bentin et al., 1996; Linkenkaer-Hansen et al., 1998). In addition, some authors suggest that the detection of visual symmetry is preferentially lateralized to the right hemisphere (Wilkinson and Halligan, 2002). Previous studies have suggested that N1 amplitude may be modulated by symmetry (Makin et al., 2012; Bertamini et al., 2018), but the association between N1 and symmetry was found to be weak, and it is also unclear if P1 shows symmetry differences as this has not been analyzed or reported in previous studies. We have no direct explanation for P1 symmetry sensitivity; however, we suggest that it may be a consequence of changing task demands (e.g., 2IFC versus 2AFC versus Yes/No or single interval), where ease of task may affect the appearance (onset) of symmetry sensitivity, i.e., in some studies, it could manifest in P1, in others in N1 (Makin et al., 2020). This may be applicable in the current design (2IFC), where, after the presentation of the first stimulus in the interval, the category of the second stimulus (symmetry or noise) is likely anticipated.

Interestingly, none of the studies examining SPN have sought to catalogue topographic changes across the SPN time window. In our previous work (Wright et al., 2018), using microstate segmentation analysis, we showed that the SPN difference wave is separable into two different microstates, and critically, that a late onsetting microstate in the SPN time window may be indicative of symmetry-sensitive mechanisms. Here, we show a topographic shift from parietal-occipital locations (of maximal ERP amplitudes) to a centrally distributed effect later in the SPN time window that is responsive to changes in luminance polarity affecting symmetry perception, which is consistent with our previous findings. For reflection symmetry, our results indicate a late SPN component that is responsive to relative cardinal angular differences (0 and 90 deg) between symmetry and luminance polarity axes, with a reduced magnitude SPN difference wave for 30 and 60 deg angular differences. This effect was more prominent when the SPN was analyzed with respect to a single noise baseline as compared to their corresponding noise baselines. To be clear, the relative angular difference is the relation of the luminance polarity axis to the off-vertical symmetry axis (jittered between +/−30 deg). Our results are in line with previous work suggesting that symmetry perception is easiest first in the vertical and then horizontal planes, but most difficult at oblique angles (Machilsen et al., 2009). However, we never presented symmetry along a horizontal axis, only the polarity axis angle was at 90 deg from the symmetry axis. Why is vertical symmetry detection affected by the changing polarity axis in a similar manner to changing the angle of the symmetry axis? One reason is that the 90 deg condition is a fully symmetric pattern (half dot-pairs are white and half dot-pairs are black), while the 0 deg condition corresponds to a fully antisymmetric stimulus (white–black dot pairs). On the other hand, the 30 and 60 deg conditions contain varying proportions of mixed-polarity pairs and same-polarity pairs, i.e., neither fully symmetric nor antisymmetric, which may explain the large increase in SPN amplitude in the late SPN window at central locations, for 0 and 90 deg conditions. It should be noted that relative cardinal angular differences (0 and 90 deg conditions) affect predominantly reflection symmetry type, but for rotation and translational symmetry SPN differences were largest when polarity-grouped 0 deg conditions were displayed. Further behavioral results indicate greater accuracy for 0 deg and not 90 deg conditions. It appears that, while the late SPN time window is topographically similar to the P300, it must also reflect some high-level process of stimulus feature-grouping/segregation.

Arguably, detection of different symmetry types involves different computational mechanisms using either bottom-up or top-down approaches. For example, reflection symmetry could be detected using pixel-by-pixel cross-correlations between the symmetric halves of the image (Barlow and Reeves, 1979; Pintsov, 1989; Gurnsey et al., 1998), early spatial mechanisms, e.g., oriented filters to compute reflection symmetry (Dakin and Watt, 1994; Osorio, 1996; Rainville and Kingdom, 2000), or complex grouping rules from which reflection-symmetry is subsequently extracted (Pashler, 1990; Wagemans et al., 1993; Labonte et al., 1995). With respect to rotational symmetry, detection is impaired compared to reflection symmetry unless the order of axes is high, e.g., 5th-order rotation symmetry (Jennings et al., 2023). Jennings et al. (2023) propose that in visual search for symmetry tasks, there are distinct mechanisms for the detection of different symmetry types: one mechanism that encodes local positional information to detect reflection- and rotation-symmetric patterns, as well as translational patterns containing few repeating sectors (one-fold symmetry). A second mechanism for detection of translational symmetry, when the number of repeating sectors is more than two, is based upon the symmetry information carried in the amplitude spectra. However, none of these models incorporate luminance polarity and/or take into account changes in the relative angular difference between symmetry and luminance polarity axes, which matters for symmetry detection in natural scenes. Our results indicate that changes in relative angular differences between luminance polarity and symmetry axes affect detection of positional symmetry, and arguably affect the salience of symmetry in a 2IFC detection task.

A novel outcome of our study was the presence of a polarity-grouping effect for noise conditions in the SPN time window – that is, a lower amplitude ERP deflection for polarity-grouped noise conditions over single-polarity noise. This effect reflects the segmentation of elements by polarity, where our noise stimuli were random in position only – half the elements were black and the other half white. While not as strong in magnitude as the SPN for symmetry, the polarity-grouping effect for noise conditions in the SPN time window appears topographically comparable to this effect, predominantly for the early time window (compare Figure 6, 8, 10 topographies for symmetry with Figure 4 topographies for polarity noise). Note the topographic changes from early to late SPN time window for the symmetry SPNs that do not occur for the noise-SPN between these time windows. The late time window may reflect a form of symmetry processing unrelated to polarity-grouping (such as position symmetry) that is not accounted for in the early time window. The implication of our polarity-grouping noise-SPN is that the SPN difference wave, considered a marker of symmetry-sensitive processing, is not always a marker for the presence of symmetry only but can reflect other structural properties of the image (e.g., luminance polarity grouping) and/or the task.

It is noteworthy that noise effects in the SPN time window, were stronger for the reflection symmetry block, substantially weaker for rotation, and missing for translation, suggesting that this noise-SPN (polarity-grouping effect) might reflect the task demands or symmetry type as it appears to scale with the accuracy of symmetry detection (e.g., reflection versus rotation versus translation, see Figure 2). It remains to be established whether the noise-SPN polarity-grouping effect is indexing structure in general or is a consequence of the 2IFC symmetry detection task. Regardless, we propose that the cognitive processes underlying all SPN effects are sensitive to other salient grouping features that form a coherent gestalt and/or modulated by saliency of symmetry (van der Helm and Treder, 2009). This conclusion is in line with our previous work highlighting the multifaceted nature of the SPN (Wright et al., 2018), showing that the early portion of the SPN time window was sensitive to the form of the stimulus (symmetry or luminance polarity grouping) while the late SPN time displayed symmetry sensitivity. Consistent with Wright et al. (2018), we suggest that the enduring nature of SPN effects may be driven by symmetry-sensitive processes in the later portion of the SPN time window, with the topographic shift suggesting a task-driven (P300-like) effect for detecting symmetry.

Further, when we analyzed the symmetry SPN with respect to a single noise baseline, we found polarity-grouping effects present in parietal-occipital electrodes for all symmetry types. This stands in contrast to our findings when SPN was analyzed using each condition’s corresponding noise baseline. Given that the magnitude of the SPN, calculated as symmetry minus noise, is affected by the noise baseline, and considering the increase in ERP amplitude for polarity-grouped over single-polarity noise conditions, processes during the SPN time window reflect not only symmetry detection but other grouping mechanisms, such as polarity-grouping. We recommend that in studies employing difference-wave analyses, such as SPN, comparing across conditions with respect to their own baselines (i.e., normalized to different baseline) should be done with caution. To put this problem another way, it is akin to measuring two different things with two different rulers and then comparing them. When different baseline conditions are used, this may mask the presence of an effect, such as polarity-grouping in the present experiment. It is therefore imperative to also compare difference-wave amplitudes with respect to a single baseline condition.

To conclude, our data demonstrate that symmetry effects can be observed as early as 100 ms in the P1 ERP component, in line with behavioral evidence suggesting symmetry detection can occur as early as 50 ms (Sharman et al., 2018). In contrast to previous work highlighting a possible role for the N1 in symmetry detection (Makin et al., 2022), we found that N1 was unresponsive to the relative angular differences between symmetry and polarity axes, and was only sensitive to grouping by luminance polarity. When examining the SPN time window, we show that it can be separated topographically into two distinct components. The SPN, when calculated with respect to the single-polarity noise condition, is modulated by polarity-grouping in the early time window for all symmetry types, yet the late time window only appeared sensitive to symmetry. The sensitivity of the SPN to changes in relative angular differences between symmetry and luminance polarity axes was present across parietal-occipital locations in the early window and shifted to a centrally distributed effect in the later time window. This effect highlights changes between cardinal (0 deg/anti-symmetry, 90 deg/full symmetry) versus other (30 and 60 deg – polarity-grouped symmetric/antisymmetric pairs) angles instead of a gradual change with increasing relative angle differences. We conclude that the luminance polarity grouping facilitates symmetry detection when symmetry is not readily salient, i.e., when the task was to detect translation and rotation, not reflection symmetry. Grouping by luminance polarity, evident also in noise only conditions, appears to be a separate process that facilitates or inhibits symmetry detection, for example in the case of oblique angle conditions.

## Supporting information

Dering et al. supplementary material Dering et al. supplementary material

## Data Availability

All data and analyses are available online at: http://hdl.handle.net/11667/235

## Supplementary material

The supplementary material for this article can be found at http://doi.org/10.1017/S0952523824000075.
