# Supplementary material for: The interaction between luminance polarity grouping and symmetry axes on the ERP responses to symmetry
Source: Vis Neurosci. 2024 Dec 16;41:E005. doi: 10.1017/S0952523824000075 (PMC11730996; doi:10.1017/S0952523824000075)
Supplement: Dering et al. supplementary material [file S0952523824000075sup001.pdf]

Supplementary Material

The interaction between luminance polarity grouping and symmetry axes on the ERP responses to symmetry

Benjamin Dering[1], Damien Wright[1,2], & Elena Gheorghiu[1,✉]

[1] *University of Stirling, Department of Psychology, Stirling, FK9 4LA, Scotland, United Kingdom*
[2] *Royal Edinburgh Hospital, Patrick Wild Centre, Division of Psychiatry, Edinburgh, EH8 9XD, Scotland, United Kingdom*

✉ Corresponding author: elena.gheorghiu@stir.ac.uk

Appendix A – Topographic ANOVA and microstate segmentation analysis

Our ERP analysis uses a mean amplitude time window approach and different scalp locations to demonstrate topographic differences between symmetry and noise conditions. However, this approach might not objectively delineate topographic differences. For example, ERP analysis of the SPN difference wave may highlight a prolonged amplitude difference between conditions, yet it cannot determine if the differences observed are due to fluctuations in field strength or represent an underlying shift in neural source generators driving the effect (Murray *et al.*, 2009).

To demonstrate objectively the existence of topographic differences within the SPN time window, we employ paired topographic ANOVAs (TANOVA) and microstate segmentation. We examined polarity effects by comparing polarity-grouped 0 deg and single-polarity symmetry conditions with respect to single-polarity noise conditions. We did this for reflection symmetry only, as this is known to generate the strongest SPN differences.

Paired topographic ANOVA (TANOVA) comparisons were performed using Cartool software (Brunet *et al.*, (2011); brainmapping.unige.ch/cartool) to determine significant periods of global dissimilarity. Global dissimilarity is an index of configuration divergence between two electric fields over time, independent of their strength (Lehmann & Skrandies, 1980). Therefore, significant divergence represents changes in scalp topography between conditions. Specifically, we conducted paired TANOVA comparisons between polarity-grouped 0 deg reflection symmetry vs. single-polarity noise and single-polarity reflection symmetry vs. single-polarity noise across the entire epoch. Given our interest in the SPN, we discuss TANOVA results around the SPN time window. The analysis identified significant windows (all p's < 0.05) of topographic dissimilarity in line with our ERP analysis (polarity-grouped 0 deg symmetry vs. single-polarity noise: 256 to 645 ms; single-polarity symmetry vs. single-polarity noise: 237 to 304 ms, 307 to 325 ms; except between 305-306 ms, for which all p's < 0.053; 384 to 722 ms, except 405 - 409 ms for which all p's < 0.058). Topographic ANOVA results are shown in Fig.S1a.

To identify whether the topographic divergences assessed by TANOVA are reflective of multiple changes in topography, we ran a microstate segmentation analysis (Pascual-Marqui *et al.*, 1995) to identify functional microstates across the two difference waves for polarity-grouped 0 deg symmetry vs. single-polarity noise and single-polarity symmetry vs. single-polarity noise conditions for reflection symmetry only.

The segmentation procedure involves a Hierarchical Clustering technique (Topographical Atomize and Agglomerate Hierarchical Clustering) performed over grand averaged ERP waveforms (Murray *et al.*, 2009; Brunet *et al.*, 2011). The topographic map at each time point is initially

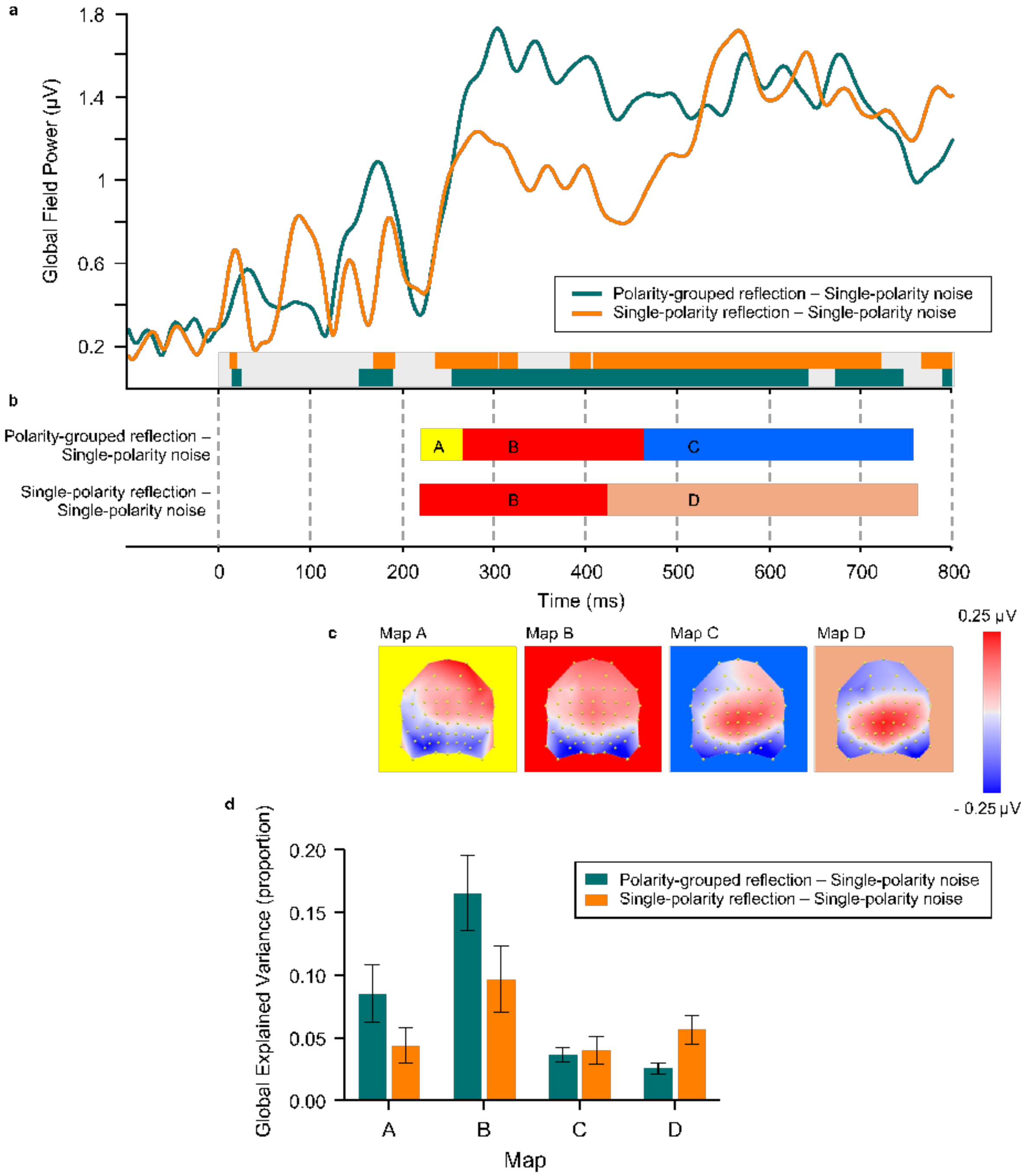

Figure S1. *(a-c) TANOVA and topographic microstate segmentation data for polarity-grouped reflection symmetry minus single-polarity noise, and single-polarity reflection symmetry minus. single-polarity noise. (a) Global Field Power (GFP) for these difference wave conditions. GFP is calculated as the spatial standard deviation of all electrodes at a given time and is a reference independent measure of the strength of electric field differences across the scalp. Green and orange horizontal bars in the inset represent periods of topographic dissimilarity identified by TANOVA. (b) The time course of microstate changes is shown, with a change in coloured bar representing a change in microstate. The coloured bars indicate which microstate represents that time period. (c) Topographic microstates derived from the segmentation procedure for four maps (A, B, C, D) which best fit the individual data within the SPN time window. Topographic maps show the head from above with nasion plotted upwards. (d) Average across participants Global Explained Variance for the polarity grouped reflection symmetry vs. single-polarity noise (green), and single-polarity reflection symmetry vs. single-polarity noise (orange) conditions plotted for each topographic microstate A, B,C and D. Note that the segmentation procedure in Fig.1b returns one map which best fits the data over each ms, but that is not to say that other maps do not fit individual participant data better (Fig 1d). This explains why for example Map A is not seen in the segmentation for single-polarity reflection symmetry minus single-polarity noise condition in Fig.1b, but the corresponding GEV is not zero in Fig.1d.*

considered a single cluster; the number of clusters/maps is then iteratively reduced into a single unique cluster, which explains the greatest variance in the data over a specific time period – and this is termed a microstate. The optimal number of microstates was determined using a meta-criterion (Brunet *et al.*, 2011), returning a model with 14 individual microstates across the two conditions during the whole epoch. We identified that only four topographic maps out of fourteen were present between the time window for analysis of the early and late SPN (250 to 600 ms; Fig.S1b). For the polarity-grouped reflection symmetry minus single-polarity noise, three unique microstates were identified, which we have labelled map A, B, and C. In the single-polarity reflection symmetry minus single-polarity noise condition only two microstates were present (with only one being unique), which we termed B and D.

We assessed the fit of these microstates to individual participants' ERP averages, by determining the proportion of Global Explained Variance (GEV) each microstate takes for each individual participant for all conditions in the time window used for the analysis of the SPN component. Note that the segmentation analysis revealed a total of fourteen individual microstate maps, each one apportioned a piece of variance in the model. Therefore, when analysing just four of these microstates the proportion of GEV can appear low. We used a competitive fitting procedure, meaning that some microstates are not present in certain individuals. Therefore, our analysis used a mixed effects model, with factors polarity (polarity grouped vs. single polarity) and microstate maps (A, B, C, D) on the GEV to compare statistical probability of each map explaining each condition. We found no significant fixed effect of polarity on GEV ($F(1,23) = 1.547$, $p = 0.226$) suggesting that microstates for polarity grouped conditions did not explain more variance than single polarity conditions. There was a significant fixed effect of microstate maps ($F(1.753,40.33) = 9.636$, $p = 0.0006$). Bonferroni-corrected pairwise comparisons revealed that only Map B differed from Map C in both polarity-grouped ($t(15) = 3.542$, $p = 0.018$, 95% CI [0.017 0.224]) and single-polarity symmetry conditions ($t(11) = 3.727$, $p = 0.02$, 95% CI [0.013 0.176]). In addition, for polarity-grouped symmetry, Map D differed from both map B ($t(14) = 3.108$, $p = 0.046$, 95% CI [0.001 0.211]) and map C ($t(13) = 3.239$, $p = 0.039$, 95% CI [0.001 0.032]). Finally, there was no significant effect between polarity and microstate maps ($F(1.612,9.674) = 2.579$, $p = 0.132$]; see Fig.S1d). Overall, while the model suggests that microstate B best fits the data for each polarity type, the segmentation clearly shows a distinct change in topography during the time window of the SPN, with the early SPN represented by maps A and B, while the late SPN is described by Maps C and D, onsetting around 450 ms (see Fig.S1c).

To help interpret the interplay between changes in neural source generators of microstates and changes in electric field strength Fig.S1 shows images of microstate segmentation alongside measures of electric field strength (Global Field Power). Global Field Power (GFP), the spatial standard deviation of all electrodes at a given time, is a reference independent measure of the strength of electric field differences across the scalp (Skrandies, 1990). A reduction in GFP without a corresponding modulation in topography can be interpreted as a reduction in the number of synchronously active neural generators (Murray *et al.*, 2008); for example, see Fig.S1a,b where Map B is present for both conditions but GFP is weaker for single-polarity symmetry minus single-polarity noise. Similarly, microstate differences between conditions can occur with no differences in GFP, as observable in the late SPN time window, with the presence of Maps C and D in the polarity-grouped and single polarity conditions, respectively.

References

Brunet D, Murray MM & Michel CM. (2011). Spatiotemporal analysis of multichannel EEG: CARTOOL. *Comput Intell Neurosci* **2011,** 813870.

Lehmann D & Skrandies W. (1980). Reference-free identification of components of checkerboard-evoked multichannel potential fields. *Electroencephalogr Clin Neurophysiol* **48,** 609-621.

Murray MM, Brunet D & Michel CM. (2008). Topographic ERP analyses: a step-by-step tutorial review. *Brain Topogr* **20,** 249-264.

Murray MM, De Lucia M, Brunet D & CM. M. (2009). Principles of topographic analyses for electrical neuroimaging. *In: Brain signal analysis: Advances in neuroelectric and neuromagnetic methods Handy TC, ed Cambridge, MA: MIT press***,** 21–54.

Pascual-Marqui RD, Michel CM & Lehmann D. (1995). Segmentation of brain electrical activity into microstates: model estimation and validation. *IEEE Trans Biomed Eng* **42,** 658-665.

Skrandies W. (1990). Global field power and topographic similarity. *Brain Topogr* **3,** 137-141.

Appendix B – One-sample t-tests for the SPN difference wave analyses

To confirm the presence of SPN difference waves, we carried out one-sample t-tests to examine whether SPN difference waves were significant from zero. These one sample t-tests were carried out following each specific ANOVA analysis examining either polarity or relative angular differences, and hence we use here the same subheadings as in our results section, for ease of reference.

Section 3.4. SPN analysis at electrodes PO7 and PO8

All SPN difference waves were significantly different from zero (all p's < 0.038) except for translation single polarity SPN (p's > 0.508) (see Fig.5c) when analysing in respect to single polarity baseline condition. In addition, when analysing with respect to their own corresponding noise baseline, the SPN was significant at PO8 only for reflection (t(23) = -3.304 , p = 0.003, d = -0.675, 95% CI [-1.113 -0.224]) and translation (t(23) = -3.098, p = 0.005, d = -0.632, 95% CI [-1.066 -0.187]), but not rotation (t(23) = -1.741, p = 0.095, d = -0.355, 95% CI [-0.765 0.061]).

Section 3.5 Noise analysis between early and late SPN time window

We also tested whether polarity-grouped minus single-polarity reflection noise conditions were different from zero at central and parietal-occipital locations (Fig.4b); this noise-SPN was found only at the parietal-occipital location in both early and late SPN windows (early: t(23) = -3.54, p = 0.002, d = -0.722 , 95% CI [-1.006 -0.264]; late: t(23) = -2.875 , p = 0.009, d = -0.587, 95% CI [-1.004 -0.164]), driven by the more negative ERP amplitude for polarity-grouped noise vs. single. For rotation-noise (Fig.4b), the noise-SPN was found in the early time window for parietal-occipital scalp locations, (t(23) = -2.174 , p = 0.04, d = -0.444, 95% CI [-0.994 -0.025]). The translation-noise showed no differences between polarity-grouped and single polarity noise (all p's > 0.131). Note the difference topographies in Fig.4b (in the main Results section) for all symmetry types are not identical to each other.

Section 3.6. Early vs. Late SPN in relation to a single noise baseline

Polarity grouping vs single polarity: One-sample t-tests confirmed that early and late time window SPNs for single and 0 deg polarity-grouped conditions, at both the central and parietal-occipital locations, were significant from zero for reflection (all p's < 0.031) and rotation (all p's <

0.048) symmetry. For translation symmetry, only 0 deg polarity-grouped was different from zero in both time windows and at both locations (all p's < 0.038), while single polarity conditions were not (all p's > 0.43).

Relative angular differences: Reflection symmetry conditions exhibited strong SPN differences over the early time window for all angles (all p's < 0.047) except for 60 deg conditions (all p's > 0.125) at both central and parietal-occipital locations. In the late time window, all angles were significant at the central locations (all p's < 0.009), while at the parietal-occipital location were not (all p's > 0.113) except for the 0 deg condition (t(23) = -2.2, p = 0.038, d = -0.449, 95% CI [-1.399 -0.043]). For rotation, in the early and late SPN time window at parietal-occipital locations, all angle conditions were not significant (all p's > 0.061). In the early time window at central locations, only the 0 and 30 deg conditions were different from zero (both p's < 0.034), whereas in the late SPN time window, all angle conditions were significantly different from zero (all p's < 0.02). Finally, translation symmetry elicited no differences for SPNs in the early or late time windows (all p's > 0.16), except in the late time window at the central electrodes for 0 deg (t(23) = 3.71, p < 0.001, d = -0.757, 95% CI [0.223 0.784]). In sum, the SPN was strongest and largest for reflection symmetry patterns, that was weaker for rotation symmetry, and weakest for translation.

Section 3.7. Early vs. Late SPN in relation to corresponding noise baselines

Polarity grouping vs single polarity: One-sample t-tests confirmed the presence of SPNs in early and late time windows at both central and parietal-occipital locations for single-polarity conditions in both reflection and rotation symmetry (all p's < 0.048), but not translation symmetry (all p's > 0.43).

For polarity-grouped conditions, all reflection-symmetry conditions were significantly different from zero in early and late time windows (all p's < 0.038). However, the SPNs for rotation symmetry in both early and late time windows was significant only at central (all p's < 0.034) but not at parietal-occipital electrodes (all p's > 0.061). For translation, none of the SPNs were significant (all p's > 0.16) except for the late time window at central electrodes (t(23) = 3.71, p = 0.001, d = -0.757, 95% CI [0.296 1.206]).

Relative angular differences: Reflection symmetry conditions exhibited strong SPN differences for all angles in both early and late time windows (all p's < 0.039), except for 30 deg, early time window, central (t(23) = 2.04, p = 0.053, d = -0.416 , 95% CI [-0.005 0.583]) and occipital locations (t(23) = -1.88, p = 0.072, d = -0.384, 95% CI [-1.152 0.054]) and 30 deg, late window, occipital location (t(23) = -1.73, p = 0.098, d = -0.449, 95% CI [-1.176 0.106]). For rotation, at central locations, 0 and 90 deg early (both p's < 0.034) and late (both p's < 0.012) windows as well as 60 deg late only (t(23) = 2.194, p = 0.039, d = -0.448, 95% CI [0.022 0.731]) showed significant differences from zero. No differences were found for 30 deg conditions (all p's > 0.176). Parietal-occipital locations exhibited strong differences only for 90 deg early (t(23) = -2.429, p = 0.023, d = -0.496, 95% CI [-0.988 -0.079]), and weak differences for 90 deg late (t(23) = -1.825, p = 0.081, d = -0.373, 95% CI [-1.225 0.077]) and 0 deg early (t(23) = -1.971, p = 0.061, d = -0.402, 95% CI [-1.003 0.024]). Finally, translation symmetry elicited no differences for SPNs in the early time window (all p's > 0.103), and in the late SPN time window only 0 deg at central locations was significant (t(23) = 3.71, p < 0.001, d = -0.757, 95% CI [0.223 0.784]) and weak evidence for an SPN for 30 deg (t(23) = 1.811, p =0.083, d = -0.37, 95% CI [-0.048 0.724]). Like the results for the single baseline conditions (section 3.6), the SPN was strongest and largest for reflection symmetry patterns, weaker for rotation symmetry, and weakest for translation.
